# Siderophore synthetase-receptor gene coevolution reveals habitat- and pathogen-specific bacterial iron interaction networks

**DOI:** 10.1126/sciadv.adq5038

**Published:** 2025-01-15

**Authors:** Shaohua Gu, Zhengying Shao, Zeyang Qu, Shenyue Zhu, Yuanzhe Shao, Di Zhang, Richard Allen, Ruolin He, Jiqi Shao, Guanyue Xiong, Alexandre Jousset, Ville-Petri Friman, Zhong Wei, Rolf Kümmerli, Zhiyuan Li

**Affiliations:** ^1^ Center for Quantitative Biology, Academy for Advanced Interdisciplinary Studies, Peking University, Beijing 100871, China.; ^2^ Peking-Tsinghua Center for Life Sciences, Academy for Advanced Interdisciplinary Studies, Peking University, Beijing 100871, China.; ^3^ Jiangsu Provincial Key Lab for Organic Solid Waste Utilization, Key Lab of Organic-based Fertilizers of China, Nanjing Agricultural University, Nanjing, P. R. China.; ^4^ Department of Quantitative Biomedicine, University of Zurich, Winterthurerstr. 190, 8057 Zurich, Switzerland.; ^5^ Department of Microbiology, University of Helsinki, 00014 Helsinki, Finland.

## Abstract

Bacterial social interactions play crucial roles in various ecological, medical, and biotechnological contexts. However, predicting these interactions from genome sequences is notoriously difficult. Here, we developed bioinformatic tools to predict whether secreted iron-scavenging siderophores stimulate or inhibit the growth of community members. Siderophores are chemically diverse and can be stimulatory or inhibitory depending on whether bacteria have or lack corresponding uptake receptors. We focused on 1928 representative *Pseudomonas* genomes and developed an experimentally validated coevolution algorithm to match encoded siderophore synthetases to corresponding receptor groups. We derived community-level iron interaction networks to show that siderophore-mediated interactions differ across habitats and lifestyles. Specifically, dense networks of siderophore sharing and competition were observed among environmental and nonpathogenic species, while small, fragmented networks occurred among human-associated and pathogenic species. Together, our sequence-to-ecology approach empowers the analyses of social interactions among thousands of bacterial strains and offers opportunities for targeted intervention to microbial communities.

## Teaser

Coevolving siderophore genes shape bacterial iron networks, unveiling the complexity of cheating across habitats and lifestyles.

## INTRODUCTION

Microbial communities populate all ecosystems on Earth from terrestrial to aquatic environments, affecting human health, agriculture, and industry (*1*–*3*). The dynamics and functioning of these communities are shaped by complex and unexplored interactions between microorganisms (*4*, *5*). As the number of sequenced microbial genomes continues to grow (*6*, *7*), there is enormous interest in developing approaches to predicting microbial interaction networks on the basis of genomic data. Such efforts are essential to obtain complete insights into community functioning as many microorganisms cannot be cultured in the laboratory (*8*), while their ecological roles could still be inferred through sequence-to-interaction mapping. Now, sequence-to-interaction mapping approaches primarily focus on metabolic interactions, with genome-scale metabolic models serving as the primary tool for establishing the pan-reactome of microbial communities (*9*, *10*). These methods infer metabolic reactions from the genome annotation of enzymes and then reconstruct a flux model to understand how microorganisms take up essential nutrients and release metabolic by-products into the environment (*11*–*13*).

Despite the significance of primary metabolism, there is increasing evidence that also other secreted compounds synthesized through secondary metabolism (*14*) play a major role in shaping microbial interactions (*15*, *16*). Nearly all microbes actively synthesize and secrete compounds to fulfill a diverse set of functions, including communication, resource scavenging, motility, and attack of and defense against competitors (*17*). Many of these secreted compounds were previously considered nonessential for microbial growth in laboratory settings but have since been shown to be critical for competitiveness in natural environments (*15*, *16*). However, sequence-to-interaction mapping has rarely been applied to secreted compounds, particularly because the synthesis and mode of action of secondary metabolites are challenging to predict.

Here, we developed a secondary metabolite sequence-to-interaction approach focusing on iron-scavenging siderophores, one of the most prevalent and diverse classes of microbial secondary metabolites (*18*). Iron is critical for microbial growth and survival because of its importance as a catalytic group in enzymes guiding key biological processes such as respiration and DNA replication (*19*). However, the concentration of bioavailable iron is typically below the required level in most habitats (*19*–*21*), and upon iron limitation, nearly all bacteria produce siderophores that efficiently chelate iron from insoluble environmental stocks (*22*, *23*). Siderophores are typically diffusible and able to chelate iron over a broad physical range (*24*). Once iron is bound, the complex is recognized and taken up by specific receptors embedded into the bacterial cell membrane (*23*). Diffusible siderophores mediate several types of social interactions. They can cooperatively be shared among contributing bacterial strains with matching receptors for the uptake of the iron-siderophore complex (*23*, *25*). Siderophores can also be deployed as competitive agents to limit access to iron if co-occurring strains lack matching receptors (*23*, *26*). Last, siderophores can be exploited by cheater bacteria that have receptors for siderophore uptake but do not pay the cost of producing siderophores themselves (*22*, *23*). Consequently, while siderophore-mediated interactions have important impacts on microbial community dynamics and functions (*27*–*30*), we still poorly understand how these interactions scale up at the network level in environmental and host-associated bacterial populations.

The aim of our study was to infer how receptors and siderophores have coevolved and use this information to develop algorithms that identify matching siderophore-receptor pairs that predict interaction networks in bacterial communities on the basis of sequence data. We previously used the genome sequences of 1928 *Pseudomonas* strains to develop bioinformatic pipelines that allowed us to predict the chemical structure of 188 pyoverdines (the main siderophores of this genus) and to identify 4547 FpvA-receptor genes (segregating into 94 groups) involved in pyoverdine uptake (*31*). Here, we capitalize on this work to develop a coevolution pairing algorithm to match the pyoverdines (key) and receptors (lock) into 47 unique lock-key groups and over 90% of these predicted interactions could be validated experimentally in vivo. Using the predicted lock-key pairs, we then reconstructed siderophore-mediated iron interaction networks among all *Pseudomonas* strains. We found that network complexity was high among strains isolated from soil-, water-, and plant-derived habitats, whereas complexity was lower among strains isolated from human-associated habitats. We further noticed that interaction networks among pathogenic species were small and loose with few pyoverdine interactions existing between strains. The opposite was the case for strains from environmental habitats. Together, the developed sequence-to-interaction mapping tool can accurately predict social interaction networks mediated by siderophores in complex bacterial communities. Our findings suggest that selection for social interactions varies across habitats and lifestyles, thus providing valuable insights into community functions and connectivity.

## RESULTS

### Three classes of pyoverdine strategies in *Pseudomonas* strains and the lock-key (receptor-synthetase) principle of coevolution

Our dataset consists of 1928 *Pseudomonas* strains, producing a total of 188 chemically different pyoverdine types and featuring 94 different receptor groups according to our recently developed bioinformatic prediction tools (*31*). Our dataset contains 1928 unique nonredundant strains as we previously deduplicated the dataset by removing strains with high phylogenic similarity and high similarity in pyoverdine synthetases. Of the 1928 nonredundant genomes, 403 are complete and 1525 are incomplete.

**Fig. 1.**
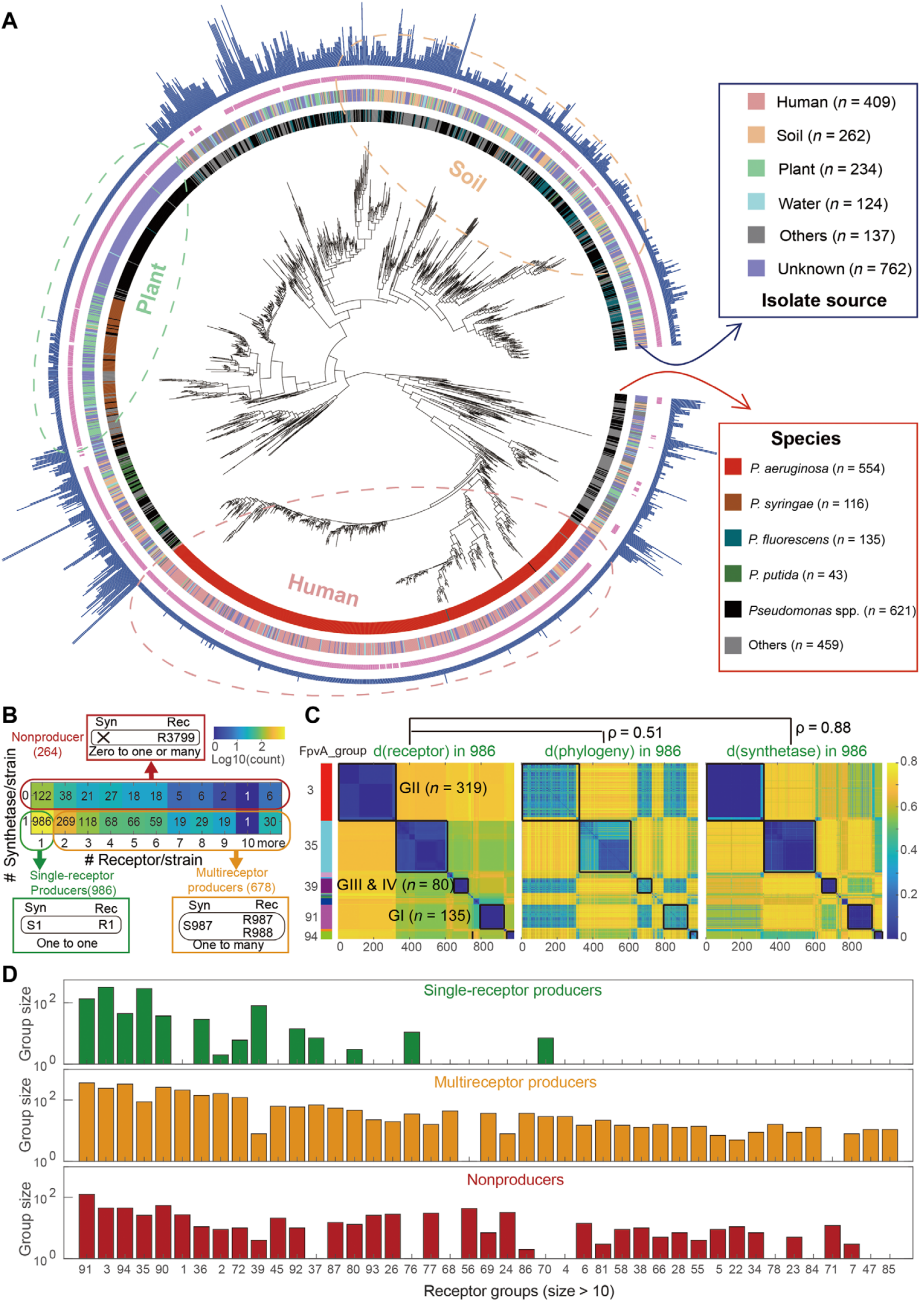
Classification of *Pseudomonas* strains and elucidation of the coevolution between pyoverdine synthetases and receptors. (**A**) Phylogenetic relationship among the 1928 *Pseudomonas* strains based on the concatenated alignment of 400 single-copy conserved genes. Starting from inside, colors in the first ring distinguish the five most prevalent species, with “Others” represent the remaining less abundant species. Colors in the second ring distinguish the four most prevalent sources of isolation. In the third ring, claret and blank regions cover strains with complete pyoverdine synthetase clusters and strains without synthetase gene clusters, respectively. In the fourth blue ring, the bar height indicates the number of FpvA receptors present in each strain. (**B**) Strains can be classified into three types by scoring the presence/absence of a synthetase cluster and counting the number receptors in each genome: (i) single-receptor producers containing one pyoverdine synthetase cluster and one FpvA receptor gene, (ii) multireceptor producers containing one pyoverdine synthetase cluster and several FpvA receptor genes, and (iii) nonproducers lacking a synthetase gene but containing at least one receptor gene. (**C**) Heatmap visualizing distances between feature sequences of the FpvA receptors and pyoverdine synthetase clusters and between FpvA feature sequences and phylogenetic genes among the 986 single-receptor producers. In all three heatmaps, the hierarchical clustering of the strains follows the one used for the FpvA feature sequences (left panel). The black squares on the heatmaps denote the five major FpvA groups. Three of these groups correspond to the receptors found among *P. aeruginosa* strains and are labeled with black text. (**D**) Forty-three largest FpvA receptor groups with more than 10 members (sorted by group size) and their frequency among single-receptor producers, multireceptor producers, and nonproducers.

We first explored the diversity of strains in terms of phylogeny, ecological habitat, and pyoverdine strategies. At the phylogenetic level, our dataset included a diverse set of *Pseudomonas* species, where *P. aeruginosa* (28.7%), *P. fluorescens* (7.0%), *P. syringae* (6.0%), and *P. putida* (2.2%) were the most abundant ones (Fig. 1A). The strains originated from diverse habitats, including human-derived habitats (21.2%), soils (13.6%), plants (12.1%), and water (6.4%), although the origin of many strains (39.5%) is unknown (Fig. 1A). While our study focuses on pyoverdine, the widespread primary siderophore of fluorescent *Pseudomonas* spp., we next checked whether secondary siderophores also occur among the 1928 strains. We found that synthetase clusters for secondary siderophores were relatively rare and occurred only in 392 strains (pyochelin: 15.1%; yersiniabactin: 3.2%; pseudomonine: 1.8%; quinolobactin: 0.15%; desferrioxamine: 0.05%; table S1). Given their rarity and the fact that secondary siderophores have lower iron affinity than pyoverdines (*32*, *33*), we can reasonably exclude the possibility that secondary siderophores have a meaningful influence on the iron networks studied here.

To assess the diversity of pyoverdine production and uptake strategies, we analyzed the absence or presence of pyoverdine synthesis clusters and counted the number of FpvA pyoverdine receptors per strain. We found three types of pyoverdine-utilization strategies (Fig. 1B). “Single-receptor producers” were the most common type (986 strains, 51.1%) and refer to strains with one pyoverdine synthesis locus and one FpvA receptor gene. “Multireceptor producers” were the second most common type (678 strains, 35.2%) and include strains with one pyoverdine synthesis cluster but multiple FpvA receptor genes. “Nonproducers” were the least common type (264 strains, 13.7%) and refer to strains that lack the pyoverdine synthesis cluster but contain at least one receptor gene. While strains can have multiple FpvA receptor genes, no strain carried more than one pyoverdine synthesis cluster. This observation is in line with the expected high costs of pyoverdine synthesis, which is based on a series of gigantic modular enzymes known as nonribosomal peptide synthetases (*34*).

On the basis of these findings, we hypothesized that in each single-receptor producer, the sole receptor present should recognize the self-produced pyoverdine to ensure fitness benefits when faced with iron limitations. Consequently, synthetase and receptor pairs should reflect molecular coevolution, where mutational alterations in the synthetase structure should select for corresponding changes in the receptor sequences to preserve the lock-key relationship and efficient iron uptake. To test this hypothesis, we focused on the 986 single-receptor producers and calculated the degree of covariation between sequence distance matrices of the receptor, the synthesis cluster, and 400 conserved genes. For the receptors (FpvA) and the synthesis cluster, we used the feature sequences that are most predictive of receptor specificity and pyoverdine molecular structure, as identified in our previous work (*31*). We found a strong correlation between the distance matrices of the receptors and the synthesis clusters (Pearson’s *r* = 0.88) and an intermediate correlation between the receptor and the conserved genes (Pearson’s *r* = 0.51) (Fig. 1C). These results provide strong evidence for an intimate coevolution between pyoverdine receptors and synthetases that greatly exceeds the baseline phylogenetic effect. Notably, we observed strong clustering patterns in the sequence space of the receptors, forming distinctive blocks that closely match with the clustering patterns of their corresponding pyoverdine synthesis clusters. Using our receptor clustering pipeline (*31*), we identified 17 receptor groups among the 986 single-receptor producers. Three out of the 17 receptor groups represent the FpvA receptors found in the human pathogen *P. aeruginosa* (labeled as GI, GII, and GIII + IV in Fig. 1C, left panel) for which the selective uptake of the corresponding pyoverdines has been demonstrated (*22*, *35*). These analyses strongly indicate that cognate receptors and synthesis genes have coevolved in single-receptor producers, resulting in one-to-one “lock-key” relationships.

Coevolutionary lock-key groups cannot directly be inferred for multireceptor producers because there is currently no method to distinguish the “self-receptor” responsible for absorbing the self-produced pyoverdine from the other FpvA receptors responsible for the uptake of heterologous pyoverdines produced by other strains. Moreover, receptor diversity seems to be much larger among multireceptor producers than among single-receptor producers. When focusing on the 43 (of 94) receptor groups with more than 10 members, we found that single-receptor producers covered only 14 of these groups (32.6%), while multireceptor producers had a much more diverse receptor coverage (41 groups, 95.3%) (Fig. 1D). Nonproducers had a similarly broad receptor coverage (34 groups, 79.1%). We also found that single-receptor producers tend to connect more compactly (mean silhouette index = 0.96 ± 0.16) than multireceptor producers (mean silhouette index = 0.78 ± 0.19) and nonproducers (mean silhouette index = 0.79 ± 0.20) in the sequence space (figs. S1 and S2), suggesting that receptors from single-receptor producers might evolutionarily be more conserved than receptors from nonproducers and multireceptor producers. Together, these observations imply that single- and multireceptor producers take on different roles in iron interaction networks.

### Development of the coevolution pairing algorithm and experimental validation for predicting iron interaction networks

The aim of this section is to establish a lock-key receptor-pyoverdine interaction map across all three strain types. A first task in this process is to identify receptors in multireceptor producers that are used to take up the self-produced pyoverdine. A first intuitive approach was to check for receptors proximate to the pyoverdine synthetase genes (solution 1), while an alternative approach was to use the lock-key pairs identified for single-receptor producers and analyze whether similar pairs occur in multireceptor strains (solution 2). Even after completing these two solutions, more than half of the receptor groups could not be paired with any pyoverdine synthetases. Specifically, solution 1 identifies putative self-receptors in 87.1% (591 of 678) of the multireceptor producers, while solution 2 could be applied to only 68.7% (466 of 678) of multireceptor strains.

**Fig. 2.**
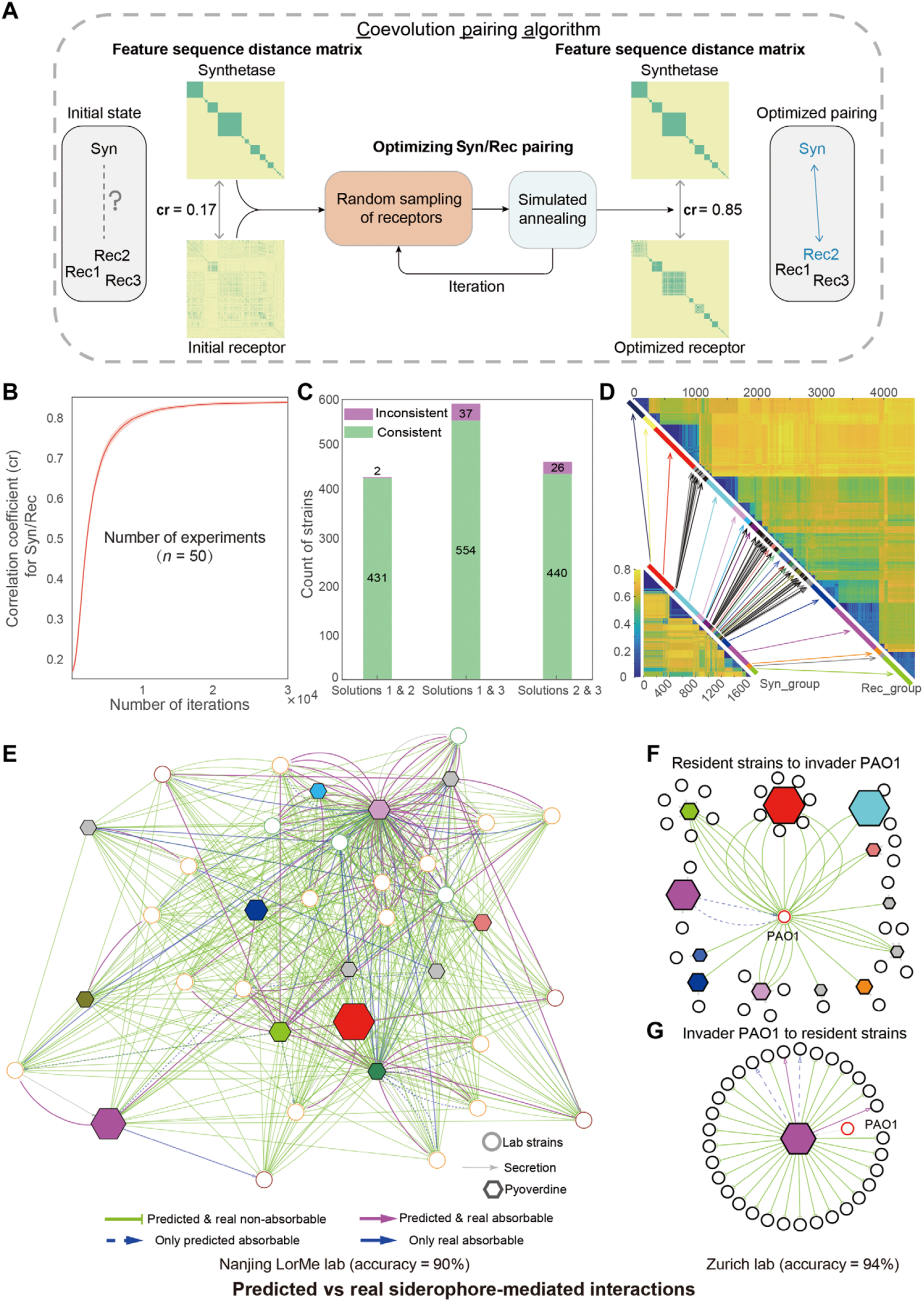
Unsupervised coevolutionary algorithm to establish a lock-key pair map for pyoverdine synthetase and receptor groups and its experimental validation. (**A**) The cartoon flowchart depicts the coevolution pairing algorithm (solution 3) to match the synthetase in each strain to its “self-receptor” on the basis of an unsupervised learning scheme that optimizes coevolutionary strength (cr values) between the feature sequence distance matrices of synthetases and matched receptors. (**B**) Progression of the cr across 3 × 10^4^ iterations based on 50 independent runs. (**C**) Consistency of identifying the same receptors as self-receptors across three different solutions. (**D**) Predicted lock-key pairs connected in sequence space: 1664 synthases (bottom left) linked to the 4547 receptors (top right). Arrows depict the 47 lock-key links between synthetase and receptor groups. The colored and black/gray-shaded lines represent groups with and without single-receptor producers, respectively. (**E**) Predicted versus observed iron interaction network among the 24 experimental strains. Each circular node represents an experimental strain, and line colors stand for single-receptor producers (green), multireceptor producers (yellow), and nonproducers (red). Hexagons represent the predicted 13 lock-key receptor-pyoverdine groups. Edges from strain nodes to lock-key nodes represent pyoverdine production, while edges from lock-key nodes to strain nodes represent utilization. Green (nonusable pyoverdine) and pink (usable pyoverdine) edges depict cases in which experimental observations match predicted interactions, while blue edges depict incorrect predictions. The pyoverdine groups that are produced by at least one single-receptor producer are colored by the respective receptor group (Fig. 1C), whereas pyoverdine groups that are exclusively produced by multireceptors are colored gray. (**F** to **G**) Predicted versus observed iron interaction networks based on data from a previous study carried out in the Zurich lab. The predicted interactions were inferred by the algorithms presented in this study, while the experimental data are taken from table S2 of Figueiredo *et al.* (*36*).

We thus developed an unsupervised learning algorithm, termed the “coevolution pairing algorithm” (solution 3), which searches for the set of synthetase-receptor combinations that maximizes coevolutionary association on the basis of feature sequence distance between the synthetase and the receptor (Fig. 2, A and B; see the “Using the coevolution relationship between synthetases and receptors to identify self-receptors in producers” section for details). The algorithm starts with a random association between synthetases and receptors, resulting in a first correlation between the two matrices [for example, correlation coefficient (cr) = 0.17]. This is then followed by an iteration process that improves the association strength of the two matrices until an optimized correlation is reached (for our dataset, cr = 0.85). Note that the cr value is expected to change depending on the dataset used. We then checked for consistency in self-receptor identification across the three solutions (Fig. 2C): (i) the receptor is within 20,000–base pair (bp) proximity to the synthetase; (ii) the lock-key pairs of single-receptor producers are applied to multireceptor producers; (iii) an unsupervised coevolution pairing algorithm. Solution 1 and solution 2 yield high levels of consistency (99.5% across 433 strains). The unsupervised solution 3 shares high consistency with solution 1 (93.7%, across 591 strains) and solution 2 (94.4%, across 466 strains), indicating that all three solutions are legitimate, with solution 3 having the advantage of being applicable to all strains.

Our coevolution pairing algorithm allocated the self-receptors of the 1664 pyoverdine-producing strain into 47 distinct lock-key groups on the basis of the receptor feature distance (Fig. 2D and fig. S3). Most self-receptors belonged to 17 lock-key groups (single-receptor producers: 986 = 100%; multireceptor producers: 572 = 84.4%), while the remaining self-receptors of multireceptor producers (106 = 15.6%) segregated into 30 additional receptor groups (fig. S3). Of the total 4547 FpvA genes detected, we identified 2883 receptors that are not self-receptors and thus possibly serve as “cheating receptors” to take up heterologous pyoverdines produced by other strains. Most of these cheating receptors (2703 = 93.8%) also segregated into the 47 lock-key groups, confirming that they could be used to exploit at least one of the 188 produced pyoverdines. The remaining cheating receptors (180 = 6.2%) could not be linked to any of the 47 lock-key groups, suggesting the existence of rare receptor groups that presumably match rare pyoverdine structures not covered by our dataset (fig. S3).

By combining the coevolution pairing algorithm and the lock-key pairing, we predicted siderophore-mediated iron interaction networks on the basis of pyoverdines that were produced and could be taken up by corresponding receptors by community members. We conducted two experiments in two different laboratories to validate predicted interactions using the same set of methods to evaluate siderophore-mediated microbial interactions. For the first validation, we used a *Pseudomonas* community from the Laboratory of Rhizosphere Microbial Ecology (LorMe) in Nanjing (China), which was originally isolated from the tomato rhizosphere (*29*). We included 24 independent strains and subjected their genomes to our bioinformatic pipelines to predict pyoverdine structures, to find all FpvA receptors (*31*), to identify self-receptors, and to allocate pyoverdines and receptors into lock-key groups. We found that these 24 strains included 4 single-receptor producers, 16 multireceptor producers, and 4 nonproducers (fig. S4) and that their self-receptors could be allocated to 13 lock-key groups (fig. S5). With this information, we predicted the pyoverdine-mediated iron interaction network between the 24 strains (Fig. 2E). For experimental validation, we confirmed the pyoverdine production status of the 20 putative producers and 4 nonproducers (fig. S4). We then followed a modified version of our previously established protocols to calculate the net effect pyoverdine has on the growth of other strains (GE_Pyo_) while controlling for the effects of other metabolites in the supernatant (*29*). This approach allowed us to obtain an experimentally derived pyoverdine-mediated iron interaction network (Fig. 2E and fig. S6). We found that 90% of the observed interactions (whether strains are stimulated or inhibited by the pyoverdines of others) matched the computationally predicted interactions from sequence data.

The second experimental validation involved strains from the Zurich (Switzerland) collection, isolated from soil and freshwater habitats (*23*). In this case, we used published experimental data from the literature (*36*). The focus of this earlier study was to test whether the opportunistic human pathogen *P. aeruginosa* PAO1 can invade natural soil and pond communities on the basis of its ability to use pyoverdine produced by the natural isolates. We used data from all the strains for which genome sequences were available (PAO1 and 33 natural isolates) to establish pyoverdine-mediated iron interaction networks (Fig. 2, F and G). We then applied our bioinformatic pipelines as explained for the Nanjing collection and found a high level of consistency (94%) between the predicted and observed pyoverdine-mediated iron interactions (Fig. 2, F and G).

Together, the two validation experiments demonstrate that siderophore-mediated microbial interactions can accurately be predicted on the basis of genome-sequence analysis using the lock-key relationship between receptor and synthetase genes. A closer investigation of the confusion matrix for the Nanjing experiment showed that the 10% inaccuracies (i.e., wrongly predicted interactions) primarily consisted of false negatives (*n* = 36, 78.3%) but few false positives (*n* = 10, 21.7%). This means that our algorithm has high specificity (97.3%) but relatively low sensitivity (67.9%). Biologically, this means that there might be additional siderophore receptor groups present among our strains, which we did not capture.

### Variation of pyoverdine-mediated iron interaction networks across habitats

**Fig. 3.**
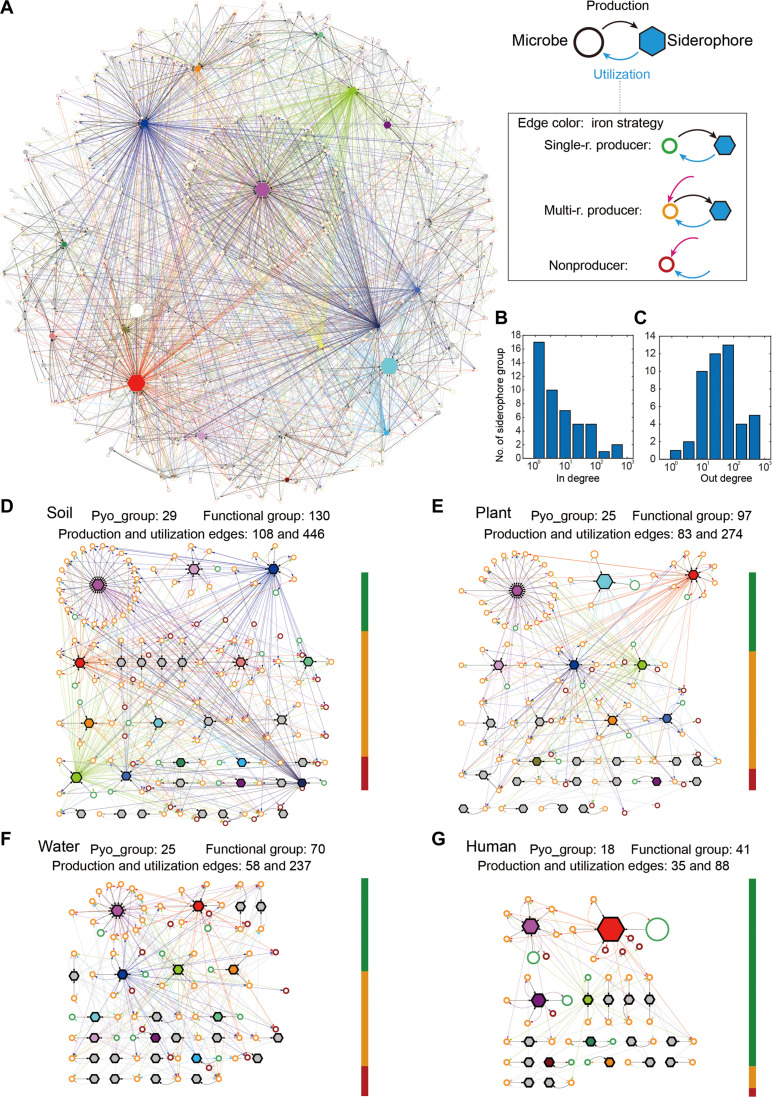
*Pseudomonas* iron interaction networks vary across habitats. (**A**) The predicted iron interaction networks mediated by pyoverdines among 1928 *Pseudomonas* strains. Circular nodes represent functional groups (i.e., strains that produce the same pyoverdine type and use the same repertoire of pyoverdines) with node size being proportional to the number of strains within this functional group. Line colors of circular nodes represent single-receptor producers (green), multireceptor producers (yellow), and nonproducers (red). Hexagonal nodes represent lock-key pyoverdine groups with node size being proportional to the number of strains using the corresponding pyoverdine. The hexagons of the 17 pyoverdine groups found among single-receptor producers are highlighted with the corresponding receptor group colors (as shown in Fig. 1C), while the pyoverdine groups that are exclusively found among multireceptor producers are depicted by gray hexagons. Edges from circular to hexagonal nodes represent pyoverdine production, while edges from hexagonal to circular nodes represent pyoverdine utilization (with edge color matching the color of the functional group). (**B**) In- and (**C**) out-degree distribution of the pyoverdine nodes. The in- and out-degrees are defined by the number of edges pointing toward (representing production) or originating from (representing utilization) a pyoverdine node. (**D** to **G**) Iron interaction networks of strains isolated from soil, plant, water and human habitats. The color bars on the right of each panel show the proportion of single-receptor producers (green), multireceptor producers (yellow), and nonproducers (red). Node symbols and colors are the same as in (A).

Following the successful validation, we applied the lock-key pairing methodology to our full dataset to construct the pyoverdine-mediated iron interaction network among all 1928 *Pseudomonas* strains (Fig. 3A). To keep traceability in such an enormous network, we allocated strains into microbial siderophore functional groups, defined as strains that produce the same pyoverdine types and can use the same repertoire of pyoverdines. Overall, the network featured 407 microbial siderophore functional groups, 47 different lock-key receptor-pyoverdine groups, 307 production edges, and 1788 utilization edges.

Our iron interaction network can be considered as a special version of a bipartite network, characterized by two types of nodes (microbial functional groups and pyoverdine groups) and two types of directional edges (utilization and production). In ecological bipartite networks such as those associated with pollination and food webs, topology plays a crucial role for ecological functions and community assemblies (*37*). The topological metrics of our network reveals considerable heterogeneity. Specifically, the in- and out-degree distribution of pyoverdine nodes is heavy tailed, indicating “hub” siderophores that are either produced or used more extensively than others (Fig. 3, B and C). This high degree of heterogeneity suggests a nonrandom network structure, likely influenced by specific eco-evolutionary forces (e.g., fierce competition for iron). We observed higher than expected yet moderate modularity (*Q*_b_ = 0.51, compared to *Q*_b_ = 0.41 in a randomized network) and nestedness (NODF = 0.15, compared to NODF = 0.04 in a randomized network). These values differ from mutualistic networks that typically feature high nestedness (e.g., pollination networks), while antagonistic networks are typically characterized by high modularity (e.g., herbivory and host-parasite networks) (*37*, *38*). Together, the iron interaction network may represent a distinct type of ecological bipartite network and stands out as one of the largest reported to date.

Next, we created separate networks for strains isolated from soil (262 strains), plant (234), water (124), and human-derived (409) habitats. We found that frequencies of the three pyoverdine strategies and network topologies varied fundamentally between the four habitats (Fig. 3, D to G). For example, among the soil-derived strains, there were 56.9% multireceptor producers, 27.5% single-receptor producers, and 15.7% nonproducers (table S2). In contrast, there were only 10.0% multireceptor producers and 4.0% nonproducers but 86.1% single-receptor producers among human-derived strains. Regarding network topologies, we observed that the number of microbial functional groups was higher for soil (130, value scaled to number of strains = 0.50), plant (97, 0.41), and water (70, 0.56) habitats than for human-related habitats (41, 0.10). Moreover, many functional groups (60.7%) exclusively occurred in a single habitat: soil (80, 23.7%), plant (56, 16.6%), water (43, 12.7%), and human (26, 7.7%), whereas only 8 functional groups (fig. S7A) and 11 pyoverdine groups (fig. S7B) were present in all four habitats. One possible reason for why the networks may differ is that the phylogenetic diversity (PD) varies across habitats. We found no evidence for such a direct association. PD was not higher in habitats with more complex networks (soil, PD = 78.1; plant, PD = 52.8; water, PD = 39.1) than in human-derived habitats (PD = 61.2) featuring simpler networks. Together, these results indicate that iron interaction networks seem to evolve differently across habitats, whereby the underlying factors shaping these differences need further investigation.

### Variation of pyoverdine-mediated iron interaction networks between pathogenic and nonpathogenic species

**Fig. 4.**
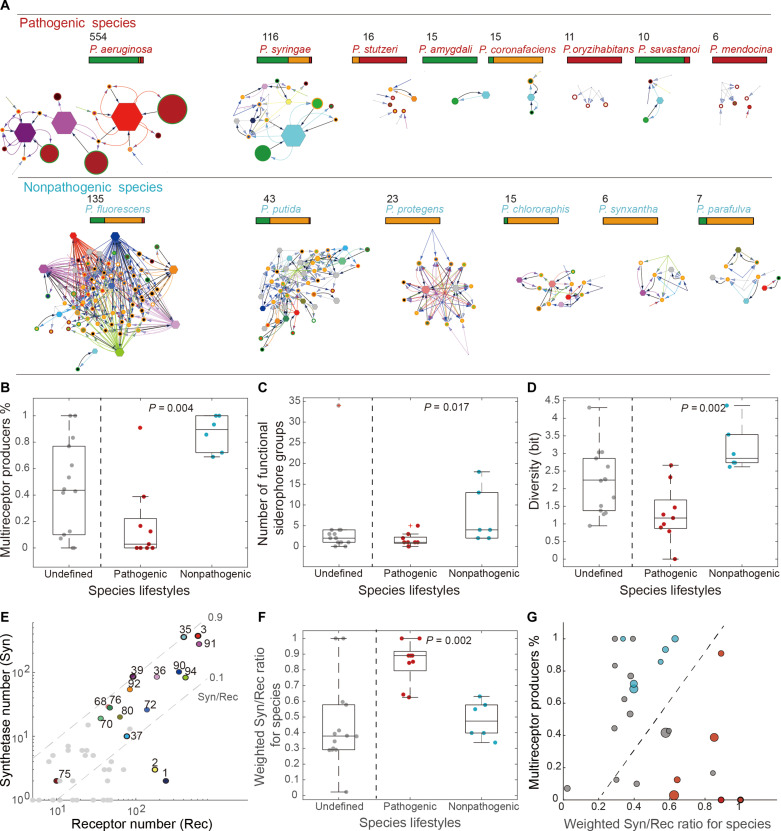
Network properties differ between pathogenic and nonpathogenic *Pseudomonas* spp. (**A**) Pyoverdine-mediated iron interaction network for pathogenic (top row) and nonpathogenic (bottom row) species with more than five strains in our database. Symbols and color codes match the ones in Fig. 3A. (**B** and **C**) Difference of the percentage of multireceptor producers and the number of the microbial functional groups between different species lifestyles. Pathogenic species are colored red, nonpathogenic species are colored blue, and species that do not have a clear role in pathogenicity are colored gray. (**D**) Difference of diversity quantified by the entropy of its functional group frequencies between different species lifestyles. (**E**) Counts of annotated receptors (*x* axis) and annotated synthetases (*y* axis) for each lock-key group. Lock-key groups are colored the same as those in Fig. 3A. The gray dotted line represents 0.1 and 0.9 Syn/Rec ratios. (**F**) Difference of the weighted Syn/Rec ratio for each species between different species lifestyles. (**G**) The percentage of multireceptor producers (*y* axis) and the weighted Syn/Rec ratio (*x* axis) for each species clearly separate pathogenic and nonpathogenic strains (segregation illustrated by the dashed line). The size of the dot represents its diversity quantified by the entropy of its functional group frequencies. Same color coding as that in (B).

*Pseudomonas* spp. do not only populate a variety of habitats but can also display diverse lifestyles. The most prominent division in lifestyle occurs between pathogenic and nonpathogenic species. Here, we explore whether iron interaction networks differ between these two lifestyles. We allocated strains to pathogenic and nonpathogenic species groups and predicted the iron interaction networks for all species with more than five strains (Fig. 4A and table S3). The most abundant pathogenic species were the human pathogen *P. aeruginosa* and the plant pathogen *P. syringae*, while the most abundant nonpathogenic environmental species were *P. fluorescens* and *P. putida*, of which many are neutral or even beneficial for hosts.

We observed multiple differences in network topologies between the two lifestyles (Fig. 4A). First, strains of pathogenic species were mostly single-receptor producers or nonproducers (Fig. 4, A and B), while strains of nonpathogenic species were primarily multireceptor producers (Fig. 4, A and B). Second, the diversity of siderophore functional groups was much lower in pathogenic species compared to nonpathogenic species (Fig. 4C and table S2). Consequently, the complexity of iron interaction networks (quantified by the entropy of their functional groups) was lower in pathogenic species than in nonpathogenic species (Fig. 4C). For example, *P. aeruginosa* (the most abundant species in our dataset, 554 strains) has a simple interaction network with only three functional groups, while *P. fluorescens* (135 strains) has a complex interaction network with 13 functional groups (table S2). Last, we calculate the synthetase count versus receptor count (termed as “Syn/Rec”) value defined as the ratio of synthetase and receptor groups present in each lock-key group (Fig. 4E). A Syn/Rec ratio near one indicates that there are no cheating receptors in this lock-key group, i.e., this pyoverdine is exclusive to its producers and cannot be used by strains not producing it. Conversely, a Syn/Rec ratio near zero means that most receptors in this lock-key group are cheating receptors, and the corresponding pyoverdine is more sharable and exploitable.

We found that the Syn/Rec ratio was much lower in nonpathogenic species than in pathogenic species (Fig. 4F). Furthermore, the Syn/Rec ratio differed substantially across lock-key groups (Fig. 4E). For example, the lock-key groups 39 (purple hexagon, present in *P. aeruginosa*) and 35 (cyan, present in several plant pathogens, Fig. 4A) had high Syn/Rec ratios of 0.93 and 0.89, respectively. In contrast, the lock-key group 94 (light green, present in *P. fluorescens*) had a much lower Syn/Rec ratio of 0.20. Collectively, the proportion of multireceptor producers and the preference for producing shareable or exclusive siderophores clearly differentiated between pathogenic and nonpathogenic species on the two-dimensional plane (Fig. 4G). Together, these results show that nonpathogenic species form open networks dominated by shareable pyoverdine lock-key groups, whereas pathogenic species form more closed networks dominated by exclusive pyoverdine lock-key groups. As in the section above, we tested whether the simpler networks observed among pathogenic species is explained by lower PD. Again, we found no evidence for such an association (nonpathogenic species: *P. fluorescens*, PD = 31.5; *P. putida*, PD = 13.3/pathogenic species: *P. aeruginosa*, PD = 71.5; *P. syringae*, PD = 18.3).

### Modeling the relationship between pyoverdine utilization strategies and community dynamics

**Fig. 5.**
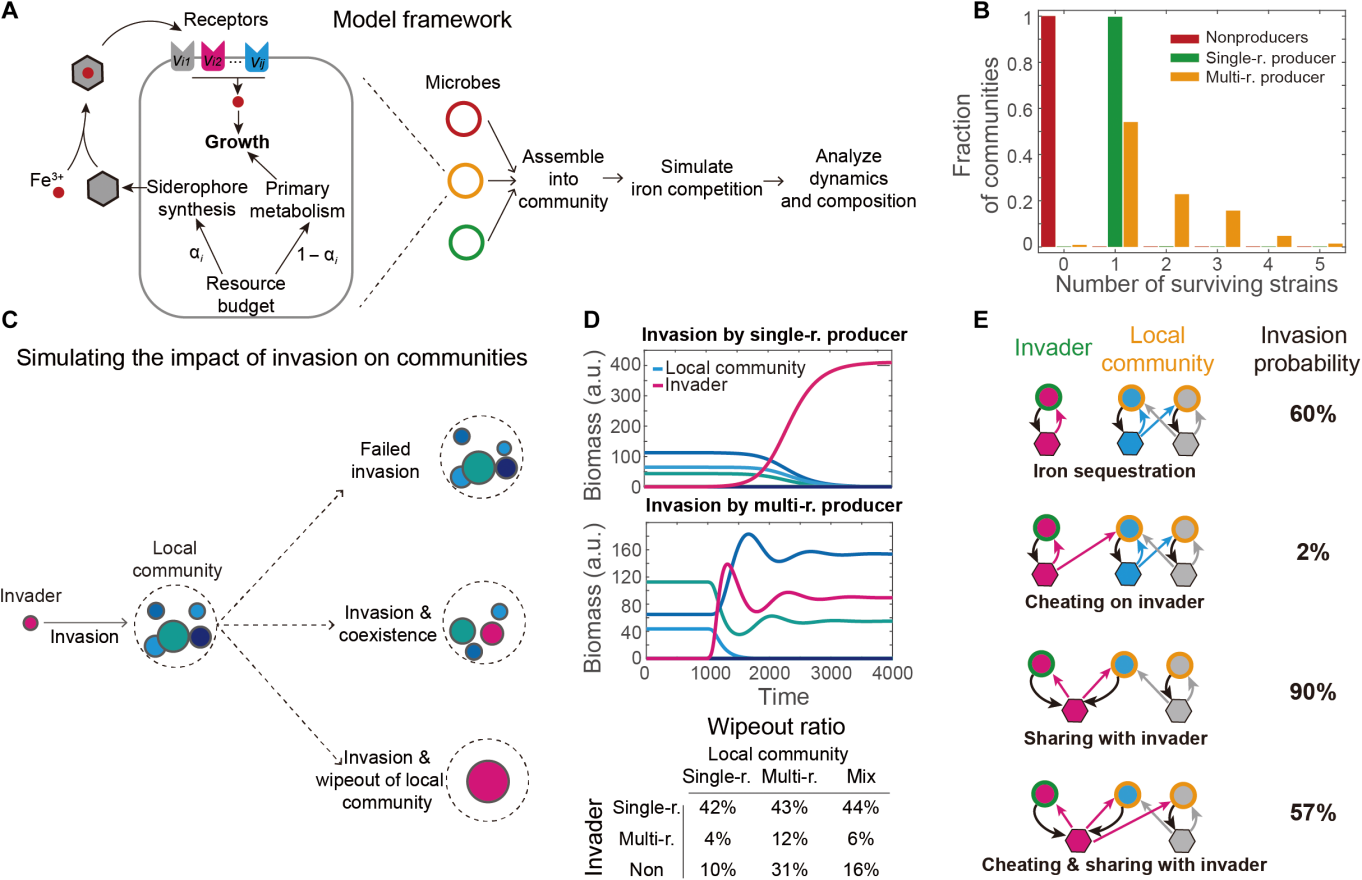
Mathematical model exploring the relationship between pyoverdine utilization strategies and community dynamics. (**A**) Schematic diagram of the iron competition model. Each microbe *i* can produce siderophores of type *j* with resource budget α_*i*_ and obtain iron by absorbing siderophore-iron complexes through corresponding receptors (fraction of receptors denoted as *v*_*ij*_). Growth rate is proportional to the total amount of absorbed iron and the fraction of resources allocated to primary metabolism, 1 − α_*i*_. In each simulation, 20 random strains are assembled into communities and then compete in a chemostat-like model until a steady state is reached. (**B**) Distribution of the number of species at the steady state for communities initiated with a single strategy type: single-receptor producers (green), multireceptor producers (yellow), and nonproducers (red). (**C**) Schematic diagram depicting the invasion scenario together with the different possible outcomes. Putative invaders are introduced at low frequency into a local community at the steady state. (**D**) Examples illustrating common invasion dynamics. (Top) A local community with three species (various blue shades) is invaded by a single-receptor producer (purple). (Middle) A local community is invaded by a multireceptor producer. (Bottom) Summary statistics showing the proportion of cases, in which invaders completely wiped out the local community as a function of their pyoverdine utilization strategy. a.u., arbitrary units. (**E**) Likelihood of a single-receptor producer to invade a local community of multireceptor producers for four different iron interaction networks. Scenarios from top to bottom show the following: (i) no overlap in siderophore utilization between the local community and the invader; (ii) the multireceptor producer from the local community can cheat on the invader’s siderophore; (iii) the invader and one multireceptor producer share the same siderophore; (iv) scenario combining (ii) and (iii). Schematics are simplifications and do not reflect the actual number of species in the local community.

To explore the connection between pyoverdine utilization strategies of individual strains and the resulting community dynamics, we built simple ecological models of siderophore-mediated ecological competition (Fig. 5A; see the Supplementary Materials for details) (*39*). Our chemostat-type model is an extension of classical resource-consumer models (*40*–*42*). It simulates the process during which various types of siderophore producers secrete their specific siderophores into the environment, which may then be used by the microbes in the system to uptake iron for their growth. We randomized key microbial parameters, such as growth budgets and resource partitioning strategies, among the individual members of the community to avoid dependencies on specific parameters. To achieve model reliability, we ran repeated simulations to gather statistical insights into community composition and dynamics. Note that our model considers bacterial agents that differ in their siderophore strategies. The strategies and situations modeled could apply to any microbes and are not limited to pseudomonads.

Our model revealed that different single-receptor producers cannot coexist, consistent with the competitive exclusion principle (*39*) and matching our observation that a high proportion of single-receptor producers was associated with simple network structures (Fig. 4A). In a well-mixed environment, competitive exclusion dictates that the number of stably coexisting species cannot exceed the number of limiting nutrients. Single-receptor producers only absorb the siderophores they produce, and their interactions with other single-receptor producers are mediated by competition for free iron. Without cross-utilization, iron remains the sole limiting nutrient, thereby allowing only a single species to persist in the community. In contrast, multireceptor producers were more likely to coexist on the basis of our model (Fig. 5B), mirroring our observation that a high proportion of multireceptor producers correlated with increased network diversity and complexity (Figs. 3 and 4A). Last, nonproducers could not exist in the absence of producers in our models.

To delve deeper into the connection between pathogenicity and pyoverdine utilization strategies, we simulated the consequences of invasion by introducing a strain (“invader”) with a higher resource budget (available for intrinsic growth and siderophore production) into a steady-state community (“local community”) (Fig. 5C). Across numerous parameters sets, we consistently observed that invasions by single-receptor producers and nonproducers tended to disrupt local communities (Fig. 5D). Successful single-receptor producers typically wiped out the local community. Conversely, successful multireceptor producers integrated themselves into and coexisted with the local community (Fig. 5D). Briefly, the more severe consequences of invasion provide a plausible explanation for the association between certain strategies and pathogenicity.

Invasion probability further depended on the pyoverdine lock-key relationship between the invader (here modeled as a single-receptor producer, e.g., matching a pathogenic lifestyle) and the local community (Fig. 5E). The probability of successful invasion was close to zero if the local community could cheat on the invader’s siderophore, which is more likely when the invader produces siderophores with low Syn/Rec ratio. Conversely, invasion probability was high if the invader could produce an exclusive siderophore or if one of the local community members produced the same siderophore as the invader (i.e., enabling cheating of the invader). Together, our modeling results reveal that the relative proportion of single- and multireceptor producers and their interactions via siderophores has fundamental consequences for community diversity and network complexity.

## DISCUSSION

Predicting interactions between microbes from sequence data offers exciting opportunities for understanding microbiome assembly and stability and may lay the foundation of biotechnological and medical microbiome interventions. While sequence-to-interaction mapping has predominantly been carried out for primary metabolism involving resource consumption, conversion, and cross-feeding, there are few approaches to reconstructing microbial interactions on the basis of secondary metabolites (antibiotics, toxins, siderophores, and surfactants) (*43*–*45*). In our paper, we offer such an approach by developing a coevolution-inspired computational approach to infer iron interaction networks mediated by pyoverdines (a class of iron-scavenging siderophores) within communities of *Pseudomonas* bacteria. Pyoverdines can both promote (through molecule sharing) and inhibit (through iron blocking) the growth of other strains depending on a molecular lock-key (receptor-pyoverdine) mechanism (*23*, *29*). Our coevolution pairing algorithm managed to pair 188 pyoverdine types and 4547 receptors into 47 lock-key groups. Our experimentally validated approach allowed us to reconstruct the iron interaction network of 1928 *Pseudomonas* strains. We found intriguing network differences between habitats (soil, plant, water, and human-derived habitats) and between microbial lifestyles (pathogenic and nonpathogenic). Large and highly connected siderophore-mediated iron interaction networks occurred among nonpathogenic environmental strains, whereas small and disconnected network dominated among pathogenic strains. These results suggest that selection pressures shaping bacterial interaction networks may differ fundamentally between habitats and lifestyles.

Our sequence-to-ecology approach underscores several challenges associated with the reconstruction of interactions driven by secondary metabolites. The first challenge is that the chemical structures of secondary metabolites are often difficult to infer from sequencing data because the metabolites are produced by nonribosomal peptide and polyketide synthesis pathways. The second challenge is to identify pyoverdine receptor genes among the many different types of siderophore receptor genes each strain has. We solved these challenges in a previous paper (*31*), where we developed approaches on the basis of feature sequences that allowed us to infer the chemical structure of 188 pyoverdines and identify 4547 pyoverdine receptor genes. The third challenge was to pair pyoverdines to matching receptors within and across strains. While coevolution analyses are a widely used computational tool, used in diverse areas ranging from ab initio protein structure to host-pathogen interaction predictions (*46*), we could not use existing algorithms, such as DCA, SCA, and Evoformer (*47*–*49*). The reason is that these classical site-based coevolution methods depend on paired sequences between which the degree of covariation is quantified, yet the existence of multireceptor producers impeded direct assignments of synthetase-receptor pairs. For this purpose, we developed an unsupervised learning algorithm (called the coevolution paring algorithm), which yielded 47 receptor-synthetase lock-key pairs. This step was essential to reconstruct iron interaction networks. Our pipeline has the potential to be applied to several other microbial traits. For example, microbial membrane receptors coevolve with phages (*50*, *51*), and pairing phages with the receptor they use for infection could provide insights into host-pathogen coevolution and, thus, bacteria-host interaction networks in natural communities.

Our sequence-to-interaction mapping together with the mathematical model yielded several important biological insights into the ecology and evolution of microbial iron interaction networks. First, multireceptor producers seem to be the glue of iron interaction networks. They harbor a large diversity of pyoverdine and receptor types, which can connect many other single- and multireceptor producers and foster the formation of large and highly connected networks. Second, networks of nonpathogenic species and communities in natural soil and water habitats were large and highly interconnected. These networks were dominated by multireceptor producers and were more open to invasion. In other words, new strains could easily integrate themselves into existing communities without much disturbance to the local community. This suggests that selection for siderophore and receptor diversity is particularly high in species-rich habitats. Third and in contrast to the above, networks of pathogenic species and communities in human-derived habitats were small and scattered and had low complexity. They were dominated by single-receptor producers with a compromised repertoire of pyoverdine and receptor types. Single-receptor producers were predicted to be the invaders with severe consequence, having a high potential to disrupt and displace the local community. Utilization of exclusive siderophore groups increases the chances of successful invasion. These results suggest that selection favors exclusive siderophores in pathogens as successful invasion is a key aspect of their lifestyle. Last, strains with a strict cheating strategy (nonproducers) occur but are relatively rare (13.6%). However, our approach yields a conservative nonproducer estimate because regulatory nonproducers that have a synthetase cluster but do not express it also occur (*22*) but cannot be detected with our approach. Given that nonproducers are fully dependent on siderophore producers, it is intuitive to understand that they are (i) more common in environmental habitats featuring many multireceptor producers with a rich pyoverdine repertoire and (ii) the best at invading multireceptor communities.

Our insights on pyoverdine utilization strategies and their consequences for community dynamics reveal ways of how siderophore-mediated interactions could be leveraged for biotechnological applications (*52*). In this context, there is great interest in using probiotic strains in agriculture to protect crops from infections by bacterial plant pathogens. There is increasing evidence that siderophore-mediated interactions play a key role in this process (*29*, *30*). For example, it was shown that plant-beneficial *Chryseobacterium* strains use their siderophores to suppress the plant pathogen *Ralstonia solanacearum* (*53*). The approach has recently been extended to human pathogens, which were found to be suppressed by exclusive siderophores from environmental *Pseudomomnas* spp. (*52*). While these studies explored siderophore interactions across the species boundaries without clear knowledge on the specific receptor setup, we here propose a strategic two-pronged design approach. First, single-receptor producers with exclusive siderophores should be designed and used as probiotics to competitively exclude pathogens in simple communities. Second, multireceptor producers with an exclusive siderophore against the pathogen and cheating receptors using the pathogen’s siderophores should be designed and used as probiotics in more complex communities. They could integrate themselves without major disruption of the local community yet still competitively exclude the pathogen via the exclusive siderophore.

In conclusion, we succeeded in developing a sequence-to-interaction mapping approach for siderophores that has a high potential to deliver deeper insights into microbial ecology. Given that iron is a key trace element that is limited in most environments, siderophore-mediated interactions are an ideal entry point for secondary metabolite analysis from sequence data. While we focused on *Pseudomonas* strains, we know that siderophore-mediated interactions occur across the genus boundaries. For example. *P. aeruginosa* has receptors to take up enterobactin produced by *Enterobacteriaceae* spp. and schizokinen produced by. *R. solanacearum* (*33*). Thus, the next step would be to apply our concepts to more diverse bacterial communities to derive microbiome-level iron-interaction maps that could further guide rational designs for biotechnological microbiota interventions.

## MATERIALS AND METHODS

### Construction of phylogeny tree

The phylogenetic tree depicted in Fig. 1A was constructed using the PhyloPhlAn3 pipeline (*54*). PhyloPhlAn is a comprehensive pipeline that encompasses the identification of phylogenetic markers, multiple sequence alignment, and the inference of phylogenetic trees. In this analysis, we used over 400 universal genes defined by PhyloPhlAn as our selected phylogeny markers. Subsequently, the taxonomic cladogram was generated using the iTOL web tool (http://huttenhower.sph.harvard.edu/galaxy/).

### Using the coevolution relationship between synthetases and receptors to identify self-receptors in producers

To establish all lock-key relationships between synthetases and receptors in their sequence spaces (see the “Variation of pyoverdine-mediated iron interaction networks across habitats” section for results), it is necessary to identify the self-receptors in each producer strain. Assuming that the strongest coevolution occurs between the synthetase and its cognate self-receptor, the pipeline of identifying self-receptors consists of following three key parts:

#### Calculation of the indel distance matrix between pyoverdine synthetase sequences

To precisly quantify the evolutionary distance between synthetases, a more accurate method than the full sequence alignment is required. In the case of module- or domain-level duplication, deletion, or insertion event, the *p*-distance [defined as the number of positions with different bases (or amino acids) in the two sequences divided by the total number of positions] between two closely related sequences can become markedly high, even for single-receptor producers whose receptor sequences belong to the same classification group. This phenomenon is common and can potentially lead to the erroneous clustering of synthetase genes. Consequently, we undertook a two-step approach to enhancing the accuracy of calculating sequence distances between synthetase genes.

*Step 1.* To address this issue, we initially conducted a global sequence alignment between any two synthetase feature sequences using the Needleman-Wunsch algorithm. Using the BLOSUM50 scoring matrix, we categorized all loci as “matched,” “similar,” or “unmatched” on the basis of their sequence similarity. Subsequently, we eliminated consecutive unmatched loci that extended for more than $L_{\text{unmatch}}$ amino acids, as these were deemed fragment mismatches resulting from module- or domain-level indels. The primary objective of this step was to mitigate the sequence distance between synthetase sequences belonging to strains within the same group. In our algorithm, the threshold $L_{\text{unmatch}}$ was set at 10 amino acids.

*Step 2.* For the remaining sequence segment, the ultimate distance, denoted as ${\text{Distance}}_{\text{syn}}$, was determined as

$${\text{Distance}}_{\text{syn}}=pdistance\times (1-p_{\text{match}})$$

Here, pdistance represents the *p*-distance of the remaining sequence, while $p_{\text{match}}$ signifies the proportion of consecutive matched loci exceeding $L_{\text{match}}$ amino acids in length. Given the fewer consecutive matched loci observed between strains belonging to different groups, this step was implemented to further accentuate the disparities in synthetase sequence distances between strains within the same group and those in different groups. In our algorithm, we set the threshold for $L_{\text{match}}$ at 10 amino acids.

#### Calculation of the correlation between synthetase and receptor sequence distance matrices

To effectively quantify the coevolutionary relationship between synthetase and receptor sequences, we used the correlation between the distance matrices of synthetic and receptor sequences. We determined self-receptor pairs by checking if the grouping of synthetase sequences matched the grouping of receptor sequences. Recognizing that the Pearson correlation coefficients between synthetase and receptor sequence distance matrices could be sensitive to minor changes in sequence distances, we executed the following two steps to robustly quantify the correlation between synthetase and receptor sequence distance matrices:

*Step 1.* We applied binarization to both the synthetase and receptor sequence distance matrices using respective thresholds, $T_{\text{syn}}$ and $T_{\text{rec}}$. The distance matrix for synthetase was the indel distance matrix described in the preceding “Calculation of the indel distance matrix between pyoverdine synthetase sequences” section, and the distance matrix for receptors was calculated by the alignment of the FpvA feature sequence (Pro^168^ to Ala^295^) (*31*). Elements exceeding the threshold were assigned a value of 1, while elements falling below the threshold were assigned a value of 0.

*Step 2.* Following binarization, it is possible that indirect connections could exist, involving intermediate strains, in addition to the direct connections between strains. To distinctly separate multiple groups, we connected all connected components that are linked to each other, directly or indirectly, as one group. Then, the distance between strains belonging to different groups was standardized to 1, while the distance between strains within the same group was consistently set to 0. This connected component approach created an unweighted network encompassing all strains. This network preserved the connection information between strains while effectively mitigating any disturbances caused by minor fluctuations in sequence distances. In our algorithm, we designated both $T_{\text{syn}}$ and $T_{\text{rec}}$ as 0.3.

#### Unsupervised coevolution pairing algorithm for identifying the self-receptor

Using the correlation calculated in the “Calculation of the correlation between synthetase and receptor sequence distance matrices” section, we developed an unsupervised algorithm that leverages random sampling and simulated annealing to identify self-receptors from a multitude of receptors in each multireceptor producer. An overview of our algorithm is presented as follows (as depicted in Fig. 3A):

*Step 1.* Initial processing.

1) In the case of N multireceptor producer strains, we initiated the process by computing the synthetase indel distances between all pairwise strains, as described in the “Calculation of the indel distance matrix between pyoverdine synthetase sequences” section. This yields a synthetase sequence distance matrix with dimensions N×N*.*

2) We randomly chose one receptor for each strain to construct the initial receptor list. Subsequently, we calculate the receptor sequence distance matrix, which is also of size N×N, on the basis of this initial receptor list.

3) We executed the connected component clustering procedure for both the synthetase and receptor sequence matrices, as described in the “Calculation of the correlation between synthetase and receptor sequence distance matrices” section. Following this, we computed the Pearson correlation coefficient between these two matrices.

*Step 2.* Random sampling. We randomly selected $N_{\text{batch}}$ strains and introduced random perturbations to the receptor numbers associated with these strains, thus generating a perturbed receptor list.

*Step 3.* Calculation of the correlation coefficient.

1) We recalculated the receptor sequence distance matrix by the perturbed receptor list.

2) The connected component clustering procedure in the “Calculation of the correlation between synthetase and receptor sequence distance matrices” section was applied to the receptor distance matrix. The correlation coefficient between the synthetase and receptor distance matrices was calculated by methods in the “Calculation of the correlation between synthetase and receptor sequence distance matrices” section.

*Step 4.* Simulated annealing. According to the correlation coefficient calculated in Step 3:

1) If the correlation coefficient is smaller than the previous value, we would accept the perturbed receptor list.

2) If the correlation coefficient failed to decrease, we would accept the change of the receptor list with a probability denoted as $P_{\text{accept}}$.

*Step 5.* We returned to Step 2 and continued the interaction, until the correlation coefficients converge or a maximial number of iterations is reached.

In each iteration, the number of randomly selected strains was adaptive. We used a smaller $N_{\text{batch}}$ when the number of iterations was limited, aiming to achieve a faster rate of correlation coefficient improvement. Conversely, when the number of iterations was extensive, we opted for a relatively larger $N_{\text{batch}}$ to introduce a wider range of perturbations. In our algorithm, the value for $N_{\text{batch}}$ was set as follows

$$N_{\text{batch}}=\left\{ \begin{array}{c} \text{Random integer from}\;\left[1,5\right],\text{iterations}<20,000\\ \text{Random integer from}\;\left[1,10\right],\text{iterations}>20,000 \end{array}\right.$$

Furthermore, the inclusion of the simulated annealing step was instrumental in preventing the algorithm from getting stuck in local optimal solutions. In our algorithm, the acceptance probability, denoted as $P_{\text{accept}}$, was configured as follows

$$P_{\text{accept}}=\text{exp}\left(\frac{\text{cr}\prime -\text{cr}}{1/S_{\text{iteration}}}\right)$$

Here, in the acceptance probability calculation, cr′ represents the correlation coefficient derived from the perturbed receptor list, cr denotes the correlation coefficient from the original receptor list, and $S_{\text{iteration}}$ signifies the current iteration count. We discarded iterations that decreased cr values and continued with those that increased cr values until an optimization plateau was reached. We predicted the self-receptor of all multireceptor producers on the basis of the final assignment. We used Pearson’s correlation coefficient for all our analyses. It is more suitable for measuring functional groups than Spearman’s rank correlation. The former is more sensitive to clustered data to measure functional coupling instead of phylogenetic associations within a functional group.

### DNA extract of *Pseudomonas* strains

We used 24 *Pseudomonas* strains that were originally isolated from tomato rhizosphere (*29*) to test the effects of pyoverdine on interactions between strains. The genomic DNA of *Pseudomonas* strains was initially extracted using the Invitrogen PureLink Genomic DNA kit. The DNA quantity and quality were tested by a NanoDrop ND-1000 Spectrophotometer (Thermo Fisher Scientific). The DNA was purified further using the Quick-DNA Miniprep Plus kit.

### Illumina HiSeq sequencing

For Illumina paired-end sequencing of each strain (*55*), at least 3 μg of genomic DNA was used for sequencing library construction. Paired-end libraries with insert sizes of ~400 bp were prepared following Illumina’s standard genomic DNA library preparation procedure. Purified genomic DNA is sheared into smaller fragments with a desired size by Covaris, and blunt ends are generated by using T4 DNA polymerase. After adding an “A” base to the 3′ end of the blunt phosphorylated DNA fragments, adapters are ligated to the ends of the DNA fragments. The desired fragments were then purified through gel electrophoresis, then selectively enriched, and amplified by polymerase chain reaction. The index tag was introduced into the adapter at the polymerase chain reaction stage as appropriate, and we did a library quality test. Last, the qualified Illumina paired-end library was used for Illumina NovaSeq 6000 sequencing (150 bp*2, Shanghai BIOZERON Co., Ltd.).

### PacBio sequencing

Whole-genome sequencing was performed by Pacific Biosciences Sequel II technology (PacBio). The DNA was made into SMRTbell libraries using the Express Template Prep Kit 2.0 from PacBio according to the manufacturer’s protocol. Samples were pooled into a single multiplexed library, and size was selected using Sage Sciences’ BluePippin, which uses the 0.75% DF Marker S1 High-Pass 6 kb–10 kb v3 run protocol and S1 marker. A size selection cutoff of 8000 (BPstart value) was used. The size-selected SMRTbell library was annealed and bound according to the SMRT Link setup and sequenced on a Sequel II.

### Genome assembly

The raw paired-end reads were trimmed and quality controlled by Trimmomatic (version 0.36, www.usadellab.org/cms/index.php?page=trimmomatic) with parameters (SLIDINGWINDOW:4:15 MINLEN:75). Clean data obtained by the above quality control processes were used for further analysis.

Raw PacBio reads were converted to fasta format with Samtools Fasta (www.htslib.org/doc/samtools.html). The Illumina data were used to evaluate the complexity of the genome and to correct the PacBio long reads. First, we used Unicycler (https://github.com/rrwick/Unicycler) to perform genome assembly with default parameters and received the optimal results of the assembly. GC depth and genome size information was calculated by custom perl scripts, which helped us to judge whether DNA samples were contaminated or not. Last, the strain genome was circularized with Circlator (http://sanger-pathogens.github.io/circlator/).

### Genome annotation

For the prokaryotic organism, we used the ab initio prediction method to get gene models for every strain. Gene models were identified using GeneMark. Then, all gene models were blastp against nonredundant (NR in NCBI) database, SwissProt (http://uniprot.org), Kyoto Encyclopedia of Genes and Genomes (www.genome.jp/kegg/), and COG (www.ncbi.nlm.nih.gov/COG) to do functional annotation by the blastp module. In addition, tRNAs were identified using tRNAscan-SE (version 1.23, http://lowelab.ucsc.edu/tRNAscan-SE), and ribosomal RNAs were determined using RNAmmer (version 1.2, https://services.healthtech.dtu.dk/services/RNAmmer-1.2/).

### Measuring the growth of *Pseudomonas* strains and their pyoverdine production

All *Pseudomonas* strains were stored at −80°C. Before the experiments, a single colony of each strain was selected randomly, grown overnight in lysogenic broth, washed three times in 0.85% NaCl, and adjusted to an optical density at 600 nm (OD_600_) of 0.5 using a spectrophotometer (SpectraMax M5, Sunnyvale, CA). To quantify the growth and siderophore production of each pseudomonad strain under iron-limited conditions, we transferred 2 ml of overnight cultures into a new 250-ml glass flask containing 150 ml of MKB medium [K_2_HPO_4_ (2.5 g l^−1^), MgSO_4_·7H_2_O (2.5 g l^−1^), glycerin (15 ml l^−1^), casamino acids (5.0 g l^−1^), pH 7.2] in threefold replication. Following 24 h of incubation at 30°C with shaking (rotary shaker set at 170 rpm), we measured growth (OD_600_) with a spectrophotometer at room temperature (SpectraMax M5, Sunnyvale, CA) and then harvested the cell-free supernatant from bacterial cultures by centrifugation (8000 rpm, 8 min at 4°C) and filtration (using a 0.22-μm filter). The supernatant was then divided into two parts for (i) measuring pyoverdine production and (ii) testing the effects of pyoverdine on interactions between *Pseudomonas* strains. Briefly, pyoverdine production levels were measured (relative fluorescence units with excitation at 400 nm and emission at 460 nm) with a spectrophotometer at room temperature (SpectraMax M5, Sunnyvale, CA).

### Testing the effects of pyoverdine on interactions between *Pseudomonas* strains

To avoid interference from other metabolites as much as possible, we adapted the method of Butaitė *et al.* (*22*) to crudely purify pyoverdine from the supernatants of 20 producers collected through the above steps. For the cross-feeding assay, we suspended each purified pyoverdine in 2 ml of Milli-Q water and passed the solution through a 0.22-μm filter.

Following the above steps to obtain 24 strains of bacterial fluid (OD_600_ = 0.5), we diluted invader precultures 100-fold into new 96-well plates and subjected them to the following three experimental conditions in threefold replication. (i) ${\text{SN}}_{\text{limited}}$: each strain individually growing in 180 μl of 10% MKB medium supplemented with 20 μl of aqueous solution of pyoverdine crude extract. (ii) ${\text{SN}}_{\text{replenished}}$: each strain individually growing in 180 μl of iron-rich 10% MKB medium supplemented with 20 μl of aqueous solution of pyoverdine crude extract (removes the effect of pyoverdine but retains the effect of other metabolites). The iron-rich condition was achieved by adding iron(III) solution (1 mM FeCl_3_·6H_2_O and 10 mM HCl) into MKB medium (final concentration equaling 50 μM). (iii) ${\text{SN}}_{\text{control}}$: each strain individually growing in 180 μl of iron-limited 10% MKB medium supplemented with 20 μl of 0.85% (w/v) NaCl instead of supernatant (control mimicking the addition of spent medium). We measured each pseudomonad strain growth (OD_600_) of each replicate after 24 h of incubation at 30°C under static conditions.

Subsequently, we calculated the effect of each producer’s pyoverdine crude extract on each pseudomonad strain growth as the growth effect, denoted as ${\text{GE}}_{\text{treatment}}$, was determined as

$${\text{GE}}_{\text{treatment}}=\left[\left({\text{SN}}_{\text{treatment}}/{\text{SN}}_{\text{control}}\right)-1\right]\times 100$$

where ${\text{SN}}_{\text{treatment}}$ is ${\text{SN}}_{\text{limited}}$ or ${\text{SN}}_{\text{replenished}}$. For this calculation, we took the average effects across the three replicates. From these measures, the net GE of pyoverdine can be measured as ${\text{GE}}_{\text{pyo}}={\text{GE}}_{\text{li}}-{\text{GE}}_{\text{re}}$. This is possible because we used the exact same supernatants for ${\text{SN}}_{\text{limited}}$ and ${\text{SN}}_{\text{replenished}}$, but pyoverdines are only important for growth in the former condition and not in the latter condition, where iron is available in excess (*29*). In principle, GE_pyo_ > 0 indicates pyoverdine-mediated facilitation. However, because there is substantial experimental variation between experimental replicates, we increased the threshold value of GE_Pyo_ > 0.05 and classified values above this threshold as positive interactions, where the receiving strain can use the respective pyoverdine for iron acquisition (interaction type 1). Conversely, GE_Pyo_ ≤ 0.05 values were classified as neutral or negative interactions, where the receiving strain cannot use the respective pyoverdine for iron acquisition (interaction type 0).

## Supplementary Materials

### This PDF file includes:

Supplementary Text Figs. S1 to S7 Tables S1 to S3 References
