# Supplementary material for: Siderophore synthetase-receptor gene coevolution reveals habitat- and pathogen-specific bacterial iron interaction networks
Source: Sci Adv. 2025 Jan 15;11(3):eadq5038. doi: 10.1126/sciadv.adq5038 (PMC11734721; doi:10.1126/sciadv.adq5038)
Supplement: Supplementary file 1 — Supplementary Text Figs. S1 to S7 Tables S1 to S3 References [file sciadv.adq5038_sm.pdf]

Science Advances
AAAS

Supplementary Materials for

Siderophore synthetase-receptor gene coevolution reveals habitat- and pathogen-specific bacterial iron interaction networks

Shaohua Gu *et al.*

Corresponding author: Zhong Wei, weizhong@njau.edu.cn; Rolf Kümmerli, rolf.kuemmerli@uzh.ch; Zhiyuan Li, zhiyuanli@pku.edu.cn

Sci. Adv. **11**, eadq5038 (2025)
DOI: 10.1126/sciadv.adq5038

This PDF file includes:

Supplementary Text
Figs. S1 to S7
Tables S1 to S3
References

Supplementary Text

Ecological model for siderophore

Our ecological model extends the traditional consumer-resource framework, by focusing on the proactive role of microorganisms in secreting siderophores to form iron-siderophore complexes, which are then selectively taken up by corresponding receptors. Due to the limited energy and protein budgets within microbial cells, we introduced two trade-offs in resource allocation: cells must decide (1) how to allocate resources between siderophore production and primary metabolism, and (2) how to distribute receptor fractions to uptake different types of siderophores. The cost of receptor synthesis is not included in our models for two reasons. First, the growth cost associated with siderophore synthesis is significantly higher, as the synthetase is more than an order of magnitude larger than the receptors. Second, in this model, the growth rate is calculated by multiplying the iron flux by the growth budget. Introducing an additional resource allocation term for receptor synthesis would add another multiplication term to the growth rate, which is mathematically equivalent to the existing model.

We assume that microbes compete in a chemostat-like environment with a constant influx and dilution rate, where iron is the sole limiting factor. The growth rate of the microbes is directly proportional to both their iron uptake rate and the resources allocated to primary metabolism.

Randomized parameters for resource allocations represent different microbial species, with multiple simulations conducted to avoid bias introduced by specific set of parameters.

Resource Competition Model for Iron Scavenging

<u>Variables in this model:</u>

Variables	Symbol
Biomass of the i-th microbe	m_i
Concentration of the siderophore of type j	$R_{\text{sid},j}$
Concentration of the siderophore-iron complex of type j	$R_{\text{com},j}$
Free iron concentration	R_{iron}

<u>Parameters used in this model (A.U.):</u>

Parameters	Parameter value
Chemostat dilution rate	$d = 0.01$
Growth coefficient	$\gamma = 20$
Siderophore-iron association constant	$k_1 = 10^{-2}$
Siderophore-iron disassociation constant	$k_2 = 10^{-7}$
Maximal transportation rate of the siderophore-iron complex	$V_m = 0.1$
Siderophore production rate per resource	$\epsilon = 0.2$
Iron supply concentration	$R_{\text{iron,supply}} = 30$

Number of species	$N_{\mathrm{spe}} = 20$
Number of siderophores	$N_{\mathrm{sid}} = 10$
Total resource budget (local community)	$\alpha_{i,0} + \sum_j \alpha_{i,j} = 1$
Total resource budget (invader)	$\alpha_{i,0} + \sum_j \alpha_{i,j} = 1.5$
Fraction of resources for primary metabolism for species i	$\alpha_{i,0}$, randomized between 0 and 1
Fraction of resources devoted to produce siderophore of type j for species i	$\alpha_{i,j}$ for $j \in \{1, \ldots, N_{\mathrm{sid}}\}$, range between 0 and 1
Fraction of receptors capable of uptaking siderophore of type j for species i	v_{ij}, range between 0 and 1

Ordinary Differential Equations:

Microbe i can produce siderophores of type j with resource budget $\alpha_{i,j}$, and uptake siderophore-iron complexes through corresponding receptors with fraction v_{ij}. Growth rate of the biomass m_i is proportional to the total amount of absorbed iron-siderophore complex ($R_{\mathrm{com},j}$) and the fraction of resources allocated to primary metabolism, $\alpha_{i,0} = 1 - \sum_j \alpha_{i,j}$:

$$\frac{\mathrm{d}m_i}{\mathrm{d}t} = \left(\sum_j \gamma \alpha_{i,0} v_{ij} V_m R_{com,j} - d\right) m_i, \quad (1)$$

$$\text{for } i = 1, \cdots, N_{\mathrm{spe}}.$$

Here, d is the dilution rate of the chemostat.

Changing rates of the concentrations of the siderophore of type j ($R_{\mathrm{sid},j}$) and the siderophore-iron complex of type j ($R_{\mathrm{com},j}$) are described as:

$$\frac{\mathrm{d}R_{\mathrm{sid},j}}{\mathrm{d}t} = -dR_{\mathrm{sid},j} + \sum_i \alpha_{i,j} \epsilon m_i - k_1 R_{\mathrm{sid},j} R_{\mathrm{iron}} + k_2 R_{\mathrm{com},j}, \quad (2)$$

$$\frac{\mathrm{d}R_{\mathrm{com},j}}{\mathrm{d}t} = -dR_{\mathrm{com},j} - \sum_i v_{ij} V_m R_{com,j} m_i + k_1 R_{\mathrm{sid}} R_{\mathrm{iron}} - k_2 R_{\mathrm{com},j}, \quad (3)$$

$$\text{where } j = 1, \cdots, N_{\mathrm{sid}}.$$

These two sets of equations described how siderophores were produced by microbes, and form complex with iron in a mass-action law fashion. The complex is then uptake by the microbes with matching receptors.

The change rate of the free iron concentration in the chemostat follows the equation below:

$$\frac{\mathrm{dR_{iron}}}{\mathrm{dt}} = \mathrm{d}\left(\mathrm{R_{ironsupply}} - \mathrm{R_{iron}}\right) - \sum_{\mathrm{j}} \mathrm{k_1 R_{sid,j} R_{iron}} + \sum_{\mathrm{j}} \mathrm{k_2 R_{com,j}} \tag{4}$$

In this equation, $R_{\mathrm{ironsupply}}$ represents the concentration of iron in the fresh medium flowing into the chemostat.

Definition of Strategies

Various species with distinct strategies is considered, including single-receptor producers, multi-receptor producers, and non-producers, differing in their $\alpha_{i,j}$ and v_{ij} values. Each different set of $\alpha_{i,j}$ and v_{ij} is considered as one species.

If species i is a single-receptor producer, we assume it only produces one type of siderophore k. Therefore, $\alpha_{i,k}$ is a random value larger than 0 and smaller than 1, and the fraction of self-receptors (the receptor intaking the siderophore produced by the species itself), v_{ik}, is 1. If species i is a multi-receptor producer, $\alpha_{i,k}$ is a random value larger than 0 and smaller than 1, and we assign the v_{ik} for self-receptors a constant value 0.2 and remaining cheating receptors (the receptors for other siderophores produced by other strains) take random values that sum up to 0.8. If species i is a non-producer, $\alpha_{i,k} = 0$ for $k \in \{1, \dots, N_{\mathrm{sid}}\}$, and v_{ij} take random value that sum to 1.

Simulation of a Community Composed Exclusively of a Single Strategic Type:

In each simulaiton, we set that a community compries 20 species with10 types of siderophores. Species satisfying the designated strategy were randomly generated with random initial inoculation densities. They then competed in a chemostat-like model for 1,400,000 time unit. The number of persisting species was recorded and analyzed. Species with biomass lower than 1 is considered as “extincted”. For communities composed by each designated strategy (single-receptor producers, multi-receptor producers, non-producers), simulations were repeated for 10,000 times.

Simulation of an invasion event within a community:

Local communities of 19 species, representing designated strategies (including single-receptor producers, multi-receptor producers, and mixtures of both), were simulated for 20,000 time unit. Simulations under each designated strategy were repeated for 5,000 times. These simulations incorporated randomized values for α_{ij}, v_{ij}, and initial biomass as previously described, ensuring a diverse range of interactions. Communities that did not go fully extinct were selected for subsequent invasion simulations.

In the invasion scenario, a species representing a pathogen (invader) was introduced into the local community with a randomly generated initial biomass between 0 and 1. The combined community, including both the local species and the invader, was then simulated for an additional 20,000 time units. Ecological outcomes of the invasion were recorded, focusing on: (1) the probability of successful invasion, defined as the invader achieving a biomass greater than 10 throughout the simulation, and (2) the likelihood of local community collapse, defined as all local species having a biomass below 1 by the end of the simulation.

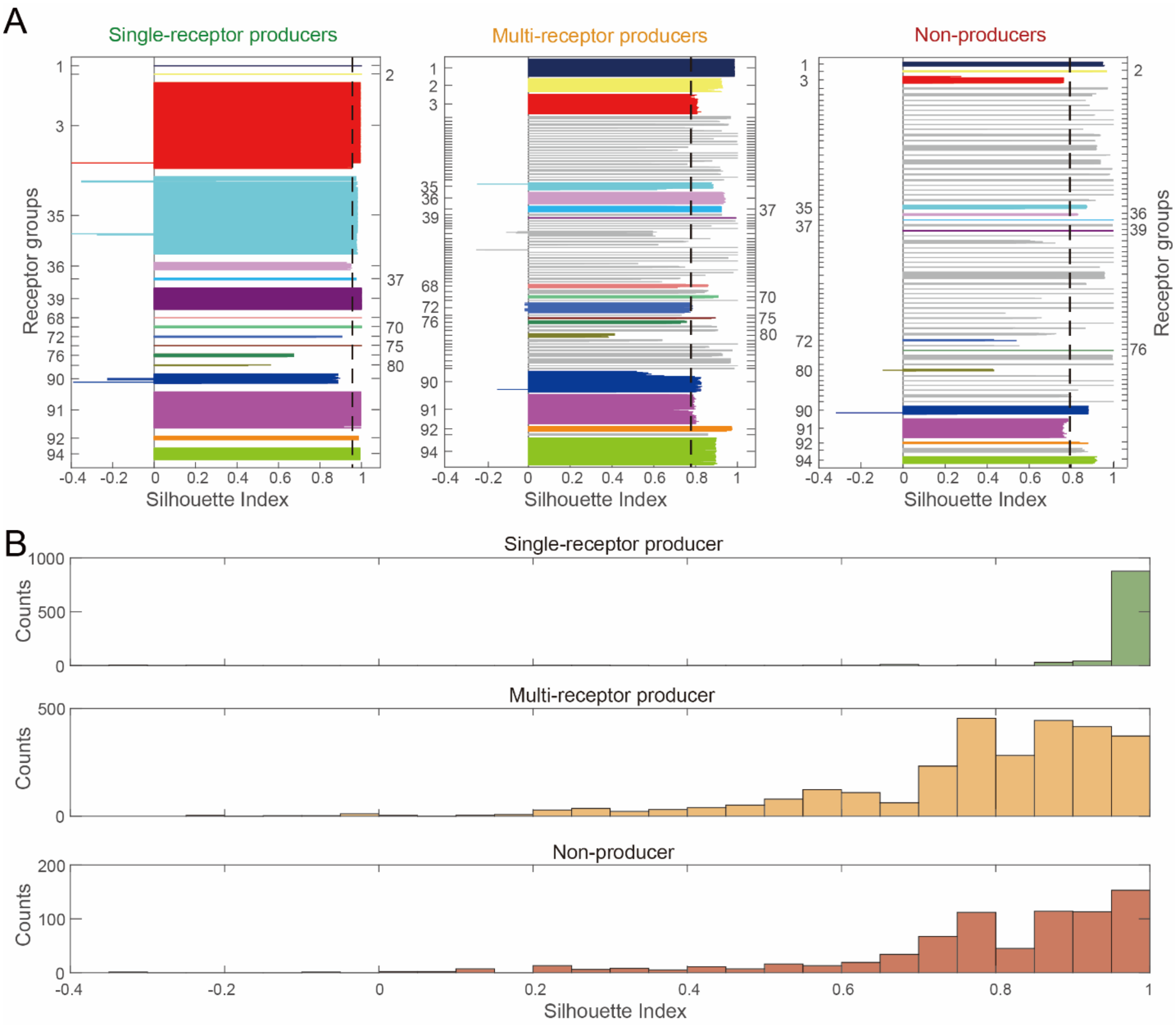

Fig. S1.

Silhouette index analysis on the compactness of all receptor groups. (**A**) Silhouette index in single-receptor producers (left panel), multi-receptor producers (middle panel) and non-producers (right panel). The silhouette index is a versatile and widely-used tool in clustering analysis because it provides a simple and interpretable way to assess the quality of clustering. It combines information about both cohesion (how similar data points within a cluster are to each other) and separation (how distinct a cluster is from other clusters). A higher average silhouette index indicates that the data points within the cluster are closely connected and the distinction between different clusters is obvious. Colors represent all the 17 receptor groups found among single-receptor producers. All other receptor groups are shown in black. The dashed vertical lines represent the average of the silhouette index across all the receptor groups within each strain class. (**B**) Histograms of the silhouette index for the three producer types, highlighting the strong clustering among single-receptor producers.

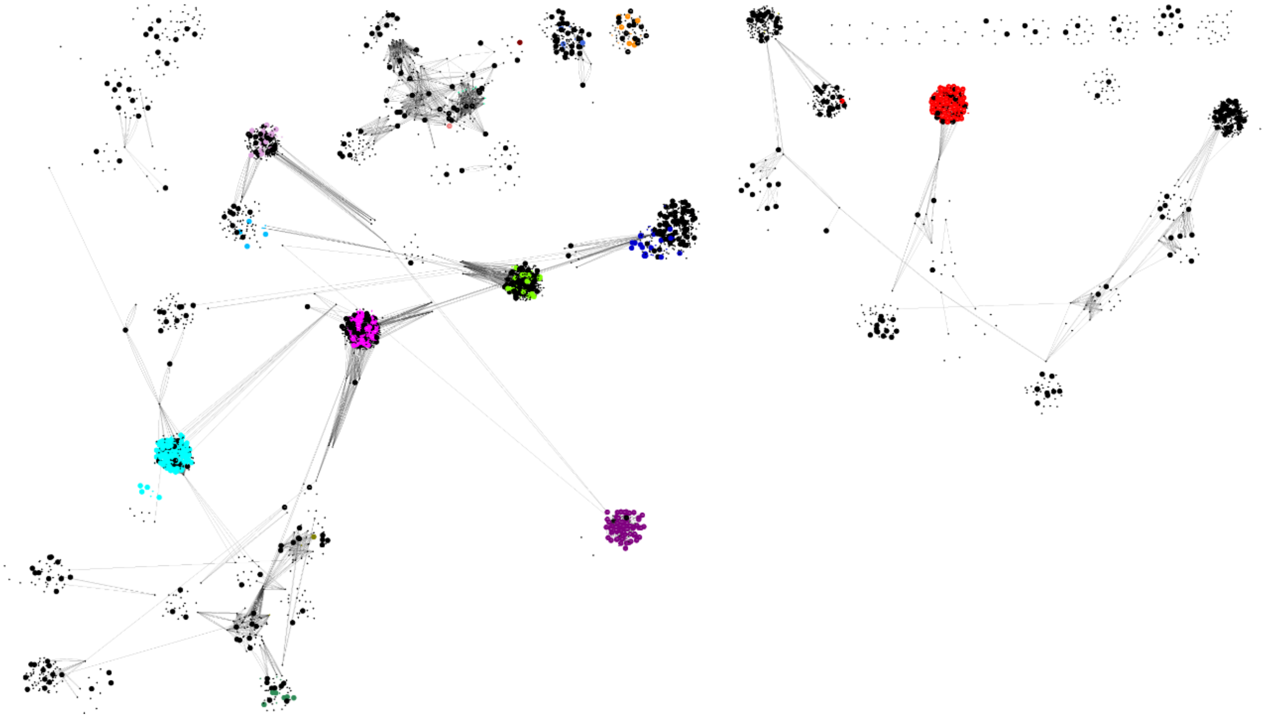

Fig. S2.

The sequence similarity network of all 4547 FpvAs receptors built by Cytoscape's edge-weighted Prefuse force-directed layout. Each FpvA is represented by a network node. Colors indicate the 17 FpvA groups found among the single-receptor producers (same color code as Fig. 1c). FpvA receptors of non-producers (small dots) and multi-receptor producers (large dots) are colored gray and black, respectively. The width of an edge represents the sequence similarity between connected nodes. Edges connecting receptors within the same group were hidden. Only edges with a similarity higher than 50% and less than 70% to other groups were displayed for each group.

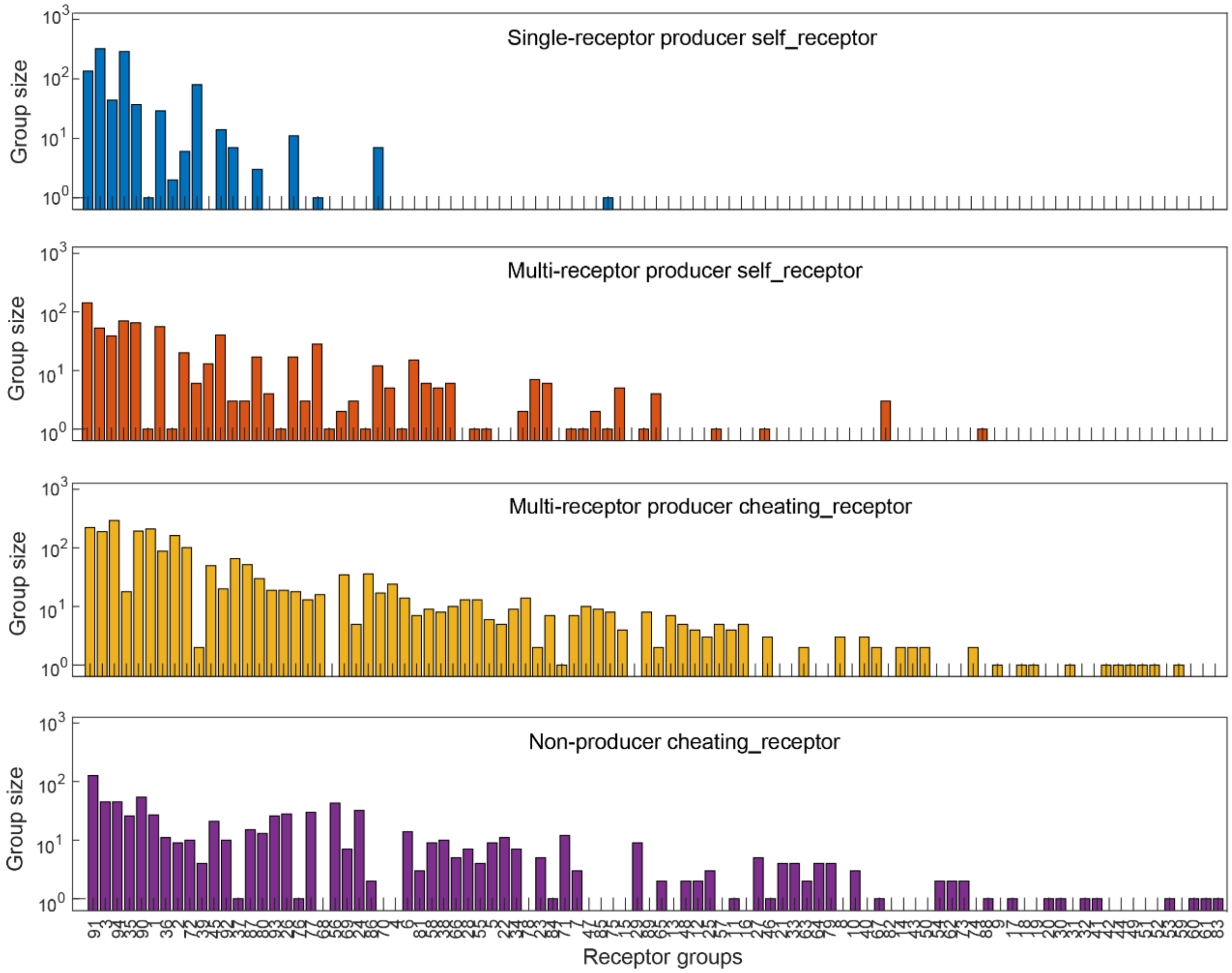

Fig. S3.

Frequency of FpvA receptor groups among three producer types. The 94 FpvA receptor groups (sorted by group size) and their frequency among single-receptor producer self_receptor, multi-receptor producer self_receptor, multi-receptor producer cheating_receptor (non-self-receptor) and non-producer cheating_receptor.

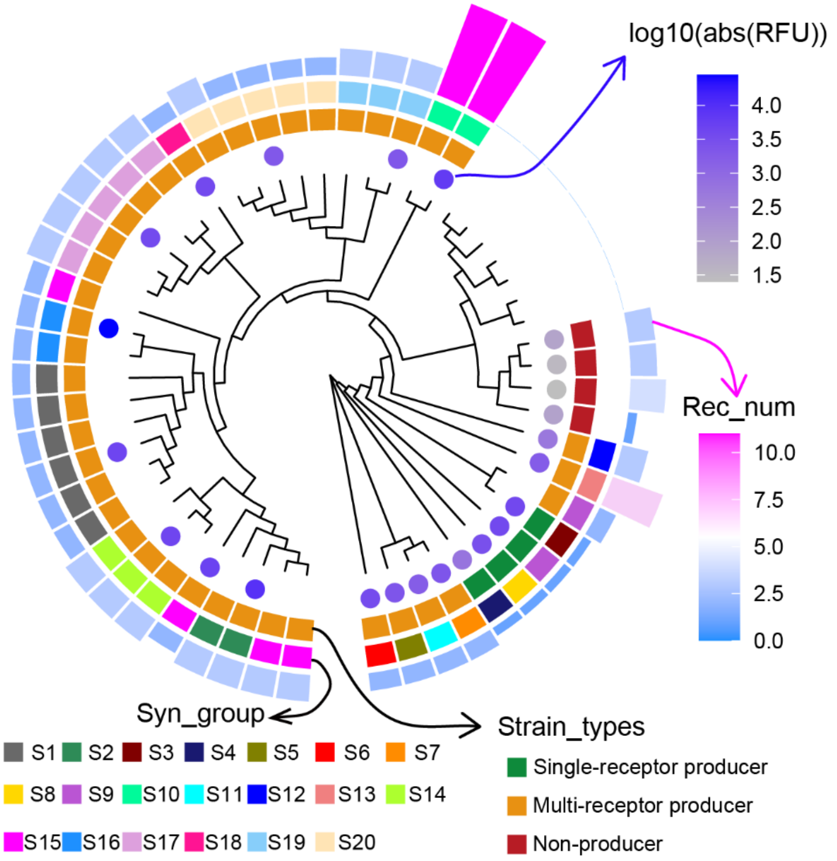

Fig. S4.

Phylogeny tree of the 55 *Pseudomonas* strains, based on concatenated alignments of 400 single-copy conserved genes. Color and bar height in the outmost ring indicate the number of FpvA receptors present in each strain. Colors in the second ring distinguish the 20 synthetase groups. Colors in the third ring highlight the three basic classes of iron-utilization strategies. To remove duplicates, we randomly selected one strain from each synthetase group to carry out the cross-feeding experiments. Colors in the innermost ring indicate the mean pyoverdine production (measured across three replicates) for the 24 experimental strains.

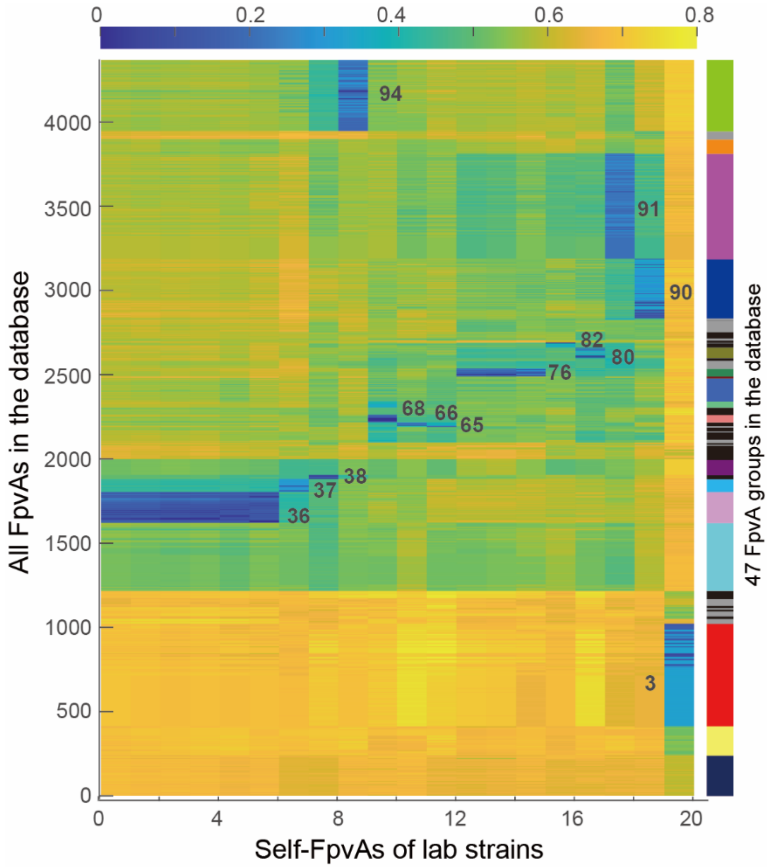

Fig. S5.

Heatmap visualization of the feature sequence distances between the self-receptors of the 20 experimental producer strains and all receptors of the 47 lock-key groups in our database. The self-receptors of the 20 experimental producer strains belong to 13 receptor groups, which are marked by black ID numbers.

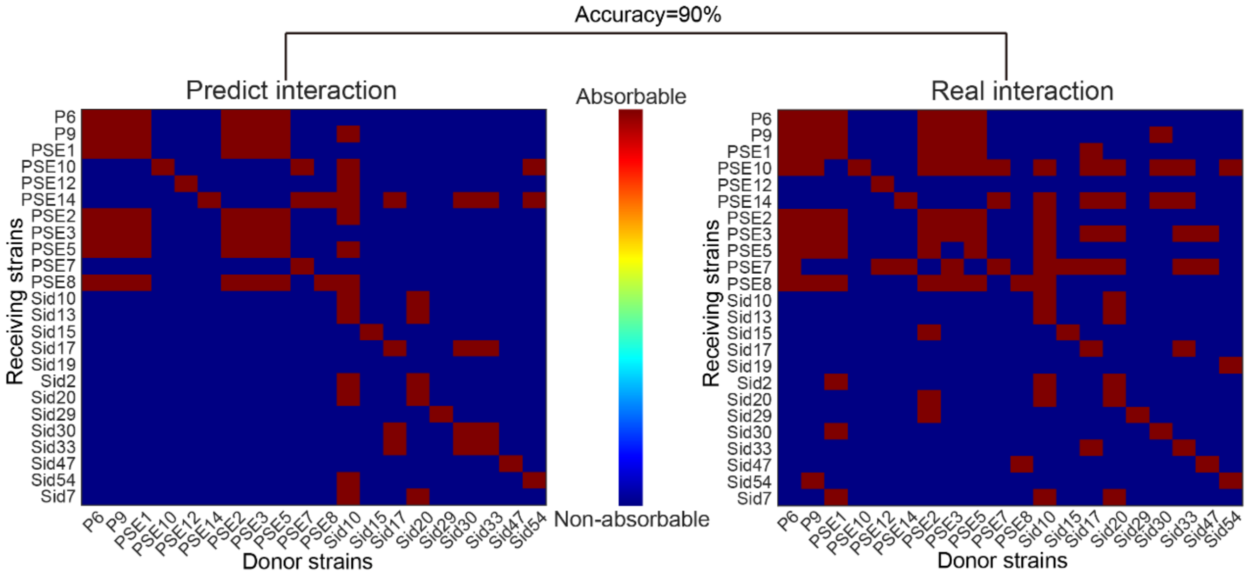

Fig. S6.

Predicted and measured pyoverdine-mediated iron interactions. Predicted pyoverdine mediated iron-interactions between laboratory strains (from Nanjing collection) based on genomic data mining and experimentally measured true pyoverdine-mediated iron interactions.

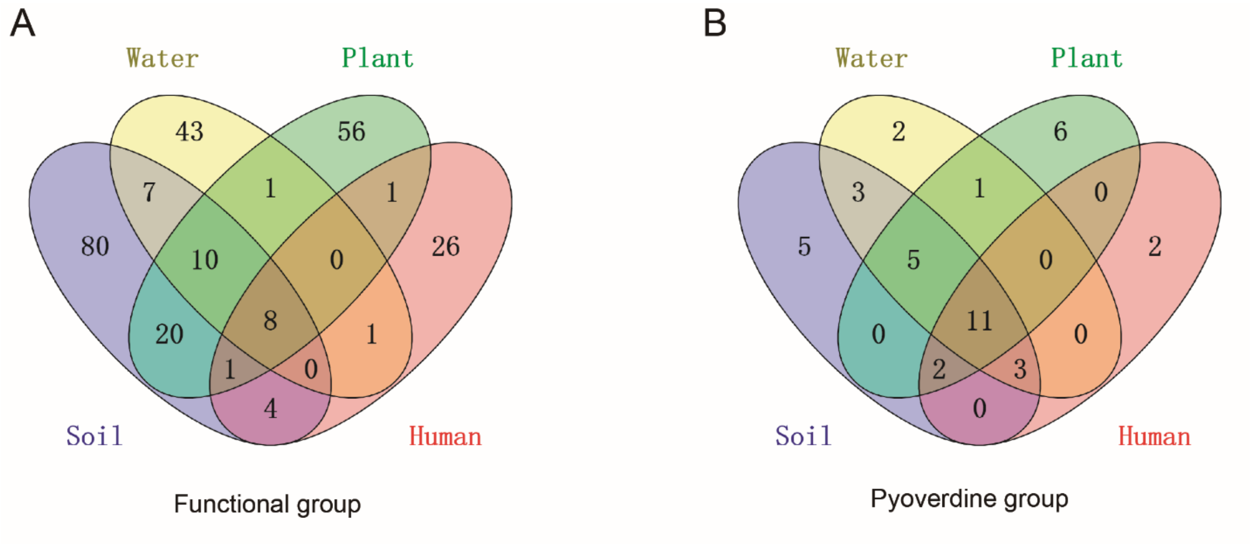

Fig. S7.

Venn diagrams of siderophore functional and pyoverdine lock-ley Groups. Venn diagrams showing the overlap in siderophore functional groups **(A)** and pyoverdine lock-key groups **(B)** of *Pseudomonas* strains isolated from different habitats.

**Other siderophores**	**counts**
Pyochelin	292
Yersiniabactin	61
Pseudomonine	35
Quinolobactin	3
Desferrioxamine	1

Table S1.

Secondary siderophores identified in the 1,928 *Pseudomonas* genomes.

	Single-receptor producer (%)	Multi-receptor producer (%)	Non-producer (%)
Soil	27.48	56.87	15.65
Plant	36.32	53.85	9.83
Water	42.74	43.55	13.71
Human	86.06	10.02	3.91

Table S2.
Distribution of *Pseudomonas* strain types in different habitats.

Species name	Strain number	Is pathogenic	Siderophore functional group number
P.sp	621	Nan	34
P.aeruginosa	554	Yes (*56*)	3
P.fluorescens	135	No (*57*)	13
P.syringae	116	Yes (*58*)	5
P.putida	43	No (*59*, *60*)	18
P.protegens	23	No (*61*)	2
P.fragi	16	Nan (*62*)	0
P.stutzeri	16	Yes (*63, 64*)	1
P.amygdali	15	Yes (*65*, *66*)	1
P.chlororaphis	15	No (*67*, *68*)	4
P.koreensis	15	Nan (*69*, *70*)	4
P.psychrotolerans	14	Nan (*71*, *72*)	1
P.brassicacearum	13	Nan (*73*)	4
P.coronafaciens	11	Yes (*74*)	1
P.oryzihabitans	11	Yes (*75*, *76*)	0
P.frederiksbergensis	10	Nan (*77*)	3
P.savastanoi	10	Yes (*78*)	1
P.moraviensis	9	Nan (*79*)	2
P.lundensis	8	Nan (*80*)	1
P.mandelii	8	Nan (*81*, *82*)	3
P.monteilii	8	Nan (*83*)	4
P.asiatica	7	nan(*84*)	1
P.parafulva	7	No (*85*)	4
P.poae	7	Nan (*86*)	2
P.asplenii	6	Yes (*79*)	2
P.helleri	6	Nan (*87*)	1
P.mendocina	6	Yes (*88*)	0
P.nitroreducens	6	Nan (*89*)	0
P.synxantha	6	No (*90*)	2

Table S3.

Classification of *Pseudomonas* species in our data set (with more than 5 strains) according to their lifestyle (pathogenic versus non-pathogenic). Nan means species that do not have clear role in pathogenicity.

REFERENCES AND NOTES

1. R. Cavicchioli, W. J. Ripple, K. N. Timmis, F. Azam, L. R. Bakken, M. Baylis, M. J. Behrenfeld, A. Boetius, P. W. Boyd, A. T. Classen, T. W. Crowther, R. Danovaro, C. M. Foreman, J. Huisman, D. A. Hutchins, J. K. Jansson, D. M. Karl, B. Koskella, D. B. Mark Welch, J. B. H. Martiny, M. A. Moran, V. J. Orphan, D. S. Reay, J. V. Remais, V. I. Rich, B. K. Singh, L. Y. Stein, F. J. Stewart, M. B. Sullivan, M. J. H. van Oppen, S. C. Weaver, E. A. Webb, N. S. Webster, Scientists' warning to humanity: Microorganisms and climate change. *Nat. Rev. Microbiol.* **17**, 569–586 (2019).

2. X. Wang, Z. Wei, K. Yang, J. Wang, A. Jousset, Y. Xu, Q. Shen, V. P. Friman, Phage combination therapies for bacterial wilt disease in tomato. *Nat. Biotechnol.* **37**, 1513–1520 (2019).

3. N. D. Sonnert, C. E. Rosen, A. R. Ghazi, E. A. Franzosa, B. Duncan-Lowey, J. A. González-Hernández, J. D. Huck, Y. Yang, Y. Dai, T. A. Rice, M. T. Nguyen, D. Song, Y. Cao, A. L. Martin, A. A. Bielecka, S. Fischer, C. Guan, J. Oh, C. Huttenhower, A. M. Ring, N. W. Palm, A host–microbiota interactome reveals extensive transkingdom connectivity. *Nature* **628**, 171–179 (2024).

4. H. K. Kuramitsu, X. He, R. Lux, M. H. Anderson, W. Shi, Interspecies interactions within oral microbial communities. *Microbiol. Mol. Biol. Rev.* **71**, 653–670 (2007).

5. A. Konopka, S. Lindemann, J. Fredrickson, Dynamics in microbial communities: Unraveling mechanisms to identify principles. *ISME J.* **9**, 1488–1495 (2015).

6. J. Handelsman, Metagenomics: Application of genomics to uncultured microorganisms. *Microbiol. Mol. Biol. Rev.* **68**, 669–685 (2004).

7. A. Almeida, A. L. Mitchell, M. Boland, S. C. Forster, G. B. Gloor, A. Tarkowska, T. D. Lawley, R. D. Finn, A new genomic blueprint of the human gut microbiota. *Nature* **568**, 499–504 (2019).

8. P. D. Schloss, J. Handelsman, Metagenomics for studying unculturable microorganisms: Cutting the Gordian knot. *Genome Biol.* **6**, 229 (2005).

9. C. Gu, G. B. Kim, W. J. Kim, H. U. Kim, S. Y. Lee, Current status and applications of genome-scale metabolic models. *Genome Biol.* **20**, 121 (2019).

10. A. V. Colarusso, I. Goodchild-Michelman, M. Rayle, A. R. Zomorrodi, Computational modeling of metabolism in microbial communities on a genome-scale. *Curr. Opin. Syst. Biol.* **26**, 46–57 (2021).

11. A. R. Pacheco, M. Moel, D. Segrè, Costless metabolic secretions as drivers of interspecies interactions in microbial ecosystems. *Nat. Commun.* **10**, 103 (2019).

12. S. Gude, M. E. Taga, Multi-faceted approaches to discovering and predicting microbial nutritional interactions. *Curr. Opin. Biotechnol.* **62**, 58–64 (2020).

13. S. Louca, M. Doebeli, Calibration and analysis of genome-based models for microbial ecology. *eLife* **4**, e08208 (2015).

14. K. A. J. Bozhüyük, L. Präve, C. Kegler, L. Schenk, S. Kaiser, C. Schelhas, Y.-N. Shi, W. Kuttenlochner, M. Schreiber, J. Kandler, M. Alanjary, T M Mohiuddin, M. Groll, G. K. A. Hochberg, H. B. Bode, Evolution-inspired engineering of nonribosomal peptide synthetases. *Science* **383**, eadg4320 (2024).

15. K. Penn, C. Jenkins, M. Nett, D. W. Udwary, E. A. Gontang, R. McGlinchey, B. Foster, A. Lapidus, S. Podell, E. E. Allen, B. S. Moore, P. R. Jensen, Genomic islands link secondary metabolism to functional adaptation in marine Actinobacteria. *ISME J.* **3**, 1193–1203 (2009).

16. K. Scherlach, C. Hertweck, Mediators of mutualistic microbe–microbe interactions. *Nat. Prod. Rep.* **35**, 303–308 (2018).

17. L. C. Vining, in *Ciba Foundation Symposium 171 - Secondary Metabolites: Their Function and Evolution* (Wiley, 2007), pp. 184–198.

18. R. He, J. Zhang, Y. Shao, S. Gu, C. Song, L. Qian, W.-B. Yin, Z. Li, Knowledge-guided data mining on the standardized architecture of NRPS: Subtypes, novel motifs, and sequence entanglements. *PLoS Comput. Biol.* **19**, e1011100 (2023).

19. S. C. Andrews, A. K. Robinson, F. Rodríguez-Quiñones, Bacterial iron homeostasis. *FEMS Microbiol. Rev.* **27**, 215–237 (2003).

20. P. W. Boyd, M. J. Ellwood, The biogeochemical cycle of iron in the ocean. *Nat. Geosci.* **3**, 675–682 (2010).

21. D. Emerson, E. Roden, B. S. Twining, The microbial ferrous wheel: Iron cycling in terrestrial, freshwater, and marine environments. *Front. Microbiol.* **3**, e00383 (2012).

22. E. Butaitė, M. Baumgartner, S. Wyder, R. Kümmerli, Siderophore cheating and cheating resistance shape competition for iron in soil and freshwater Pseudomonas communities. *Nat. Commun.* **8**, 414 (2017).

23. J. Kramer, Ö. Özkaya, R. Kümmerli, Bacterial siderophores in community and host interactions. *Nat. Rev. Microbiol.* **18**, 152–163 (2020).

24. G. E. Leventhal, M. Ackermann, K. T. Schiessl, Why microbes secrete molecules to modify their environment: the case of iron-chelating siderophores. *J. R. Soc. Interface* **16**, 20180674 (2019).

25. S. A. West, S. P. Diggle, A. Buckling, A. Gardner, A. S. Griffin, The social lives of microbes. *Annu. Rev. Ecol. Evol. Syst.* **38**, 53–77 (2007).

26. M. Miethke, M. A. Marahiel, Siderophore-based iron acquisition and pathogen control. *Microbiol. Mol. Biol. Rev.* **71**, 413–451 (2007).

27. Y. Seyoum, K. Baye, C. Humblot, Iron homeostasis in host and gut bacteria – A complex interrelationship. *Gut Microbes* **13**, 1–19 (2021).

28. U. E. Schaible, S. H. E. Kaufmann, Iron and microbial infection. *Nat. Rev. Microbiol.* **2**, 946–953 (2004).

29. S. Gu, Z. Wei, Z. Shao, V. P. Friman, K. Cao, T. Yang, J. Kramer, X. Wang, M. Li, X. Mei, Y. Xu, Q. Shen, R. Kümmerli, A. Jousset, Competition for iron drives phytopathogen control by natural rhizosphere microbiomes. *Nat. Microbiol.* **5**, 1002–1010 (2020).

30. S. Gu, T. Yang, Z. Shao, T. Wang, K. Cao, A. Jousset, V.P. Friman, C. Mallon, X. Mei, Z. Wei, Y. Xu, Q. Shen, T. Pommier, Siderophore-mediated interactions determine the disease suppressiveness of microbial consortia. *mSystems* **5**, e00811-19 (2020).

31. S. Gu, Y. Shao, K. Rehm, L. Bigler, D. Zhang, R. He, J. Shao, A. Jousset, V.-P. Friman, Z. Wei, R. Kümmerli, Z. Li, From sequence to molecules: Feature sequence-based genome mining uncovers the hidden diversity of bacterial siderophore pathways. bioRxiv [Preprint]. 2023. https://doi.org/10.1101/2023.10.30.564663.

32. P. Cornelis, Iron uptake and metabolism in pseudomonads. *Appl. Microbiol. Biotechnol.* **86**, 1637–1645 (2010).

33. R. Kümmerli, Iron acquisition strategies in pseudomonads: Mechanisms, ecology, and evolution. *Biometals* **36**, 777–797 (2023).

34. K. A. J. Bozhüyük, F. Fleischhacker, A. Linck, F. Wesche, A. Tietze, C. P. Niesert, H. B. Bode, De novo design and engineering of non-ribosomal peptide synthetases. *Nat. Chem.* **10**, 275–281 (2018).

35. J. M. Meyer, A. Stintzi, D. de Vos, P. Cornelis, R. Tappe, K. Taraz, H. Budzikiewicz, Use of siderophores to type pseudomonads: The three Pseudomonas aeruginosa pyoverdine systems. *Microbiology* **143**, 35–43 (1997).

36. A. R. T. Figueiredo, Ö. Özkaya, R. Kümmerli, J. Kramer, Siderophores drive invasion dynamics in bacterial communities through their dual role as public good versus public bad. *Ecol. Lett.* **25**, 138–150 (2022).

37. E. Thébault, C. Fontaine, Stability of ecological communities and the architecture of mutualistic and trophic networks. *Science* **329**, 853–856 (2010).

38. J. Bascompte, P. Jordano, C. J. Melián, J. M. Olesen, The nested assembly of plant–animal mutualistic networks. *Proc. Natl. Acad. Sci. U.S.A.* **100**, 9383–9387 (2003).

39. S. Jiqi, L. Yinxiang, L. Jingyuan, G. Shaohua, L. Zhiyuan, Siderophore piracy promotes dynamical coexistence in microbial community. bioRxiv [Preprint]. 2023. https://doi.org/10.1101/2023.11.21.568182.

40. D. Tilman, *Resource Competition and Community Structure* (Princeton University Press, 1982).

41. V. Dubinkina, Y. Fridman, P. P. Pandey, S. Maslov, Multistability and regime shifts in microbial communities explained by competition for essential nutrients. *eLife* **8**, e49720 (2019).

42. T. Taillefumier, A. Posfai, Y. Meir, N. S. Wingreen, Microbial consortia at steady supply. *eLife* **6**, e22644 (2017).

43. M. C. B. Tsilimigras, A. A. Fodor, Compositional data analysis of the microbiome: Fundamentals, tools, and challenges. *Ann. Epidemiol.* **26**, 330–335 (2016).

44. S. Weiss, W. van Treuren, C. Lozupone, K. Faust, J. Friedman, Y. Deng, L. C. Xia, Z. Z. Xu, L. Ursell, E. J. Alm, A. Birmingham, J. A. Cram, J. A. Fuhrman, J. Raes, F. Sun, J. Zhou, R. Knight, Correlation detection strategies in microbial data sets vary widely in sensitivity and precision. *ISME J.* **10**, 1669–1681 (2016).

45. K. Faust, J. Raes, Microbial interactions: From networks to models. *Nat. Rev. Microbiol.* **10**, 538–550 (2012).

46. H. M. Chen, W. Guo, J. Shen, L. Wang, J. N. Song, Structural principles analysis of host-pathogen protein-protein interactions: A structural bioinformatics survey. *IEEE Access* **6**, 11760–11771 (2018).

47. J. Greensmith, J. Feyereisl, U. Aickelin, The DCA: SOMe comparison. *Evol. Intell.* **1**, 85–112 (2008).

48. S. Mirjalili, SCA: A sine cosine algorithm for solving optimization problems. *Knowl.-Based Syst.* **96**, 120–133 (2016).

49. J. Jumper, R. Evans, A. Pritzel, T. Green, M. Figurnov, O. Ronneberger, K. Tunyasuvunakool, R. Bates, A. Žídek, A. Potapenko, A. Bridgland, C. Meyer, S. A. A. Kohl, A. J. Ballard, A. Cowie, B. Romera-Paredes, S. Nikolov, R. Jain, J. Adler, T. Back, S. Petersen, D. Reiman, E. Clancy, M. Zielinski, M. Steinegger, M. Pacholska, T. Berghammer, S. Bodenstein, D. Silver, O. Vinyals, A. W. Senior, K. Kavukcuoglu, P. Kohli, D. Hassabis, Highly accurate protein structure prediction with AlphaFold. *Nature* **596**, 583–589 (2021).

50. H. G. Hampton, B. N. J. Watson, P. C. Fineran, The arms race between bacteria and their phage foes. *Nature* **577**, 327–336 (2020).

51. K. E. Kortright, B. K. Chan, J. L. Koff, P. E. Turner, Phage Therapy: A renewed approach to combat antibiotic-resistant bacteria. *Cell Host Microbe* **25**, 219–232 (2019).

52. V. Vollenweider, K. Rehm, C. Chepkirui, M. Pérez-Berlanga, M. Polymenidou, J. Piel, L. Bigler, R. Kümmerli, Antimicrobial activity of iron-depriving pyoverdines against human opportunistic pathogens. bioRxiv [Preprint]. 2023. https://doi.org/10.1101/2023.07.18.549568.

53. K. Rehm, V. Vollenweider, S. Gu, V. P. Friman, R. Kümmerli, Z. Wei, L. Bigler, Chryseochelins—Structural characterization of novel citrate-based siderophores produced by plant protecting Chryseobacterium spp. *Metallomics* **15**, mfad008 (2023).

54. F. Asnicar, A. M. Thomas, F. Beghini, C. Mengoni, S. Manara, P. Manghi, Q. Zhu, M. Bolzan, F. Cumbo, U. May, J. G. Sanders, M. Zolfo, E. Kopylova, E. Pasolli, R. Knight, S. Mirarab, C. Huttenhower, N. Segata, Precise phylogenetic analysis of microbial isolates and genomes from metagenomes using PhyloPhlAn 3.0. *Nat. Commun.* **11**, 2500 (2020).

55. Y. Wang, Z. Sha, X. Ren, A new species of Orchomenella (Amphipoda, Tryphosidae) described from hydrothermal vent in the Okinawa Trough, Northwest Pacific. *ZooKeys* **1184**, 261–271 (2023).

56. S. P. Diggle, M. Whiteley, Microbe Profile: Pseudomonas aeruginosa: Opportunistic pathogen and lab rat. *Microbiology* **166**, 30–33 (2020).

57. B. S. Scales, R. P. Dickson, J. J. LiPuma, G. B. Huffnagle, Microbiology, genomics, and clinical significance of the Pseudomonas fluorescens species complex, an unappreciated colonizer of humans. *Clin. Microbiol. Rev.* **27**, 927–948 (2014).

58. D. L. Arnold, G. M. Preston, Pseudomonas syringae: Enterprising epiphyte and stealthy parasite. *Microbiology* **165**, 251–253 (2019).

59. C. L. Patten, B. R. Glick, Role of Pseudomonas putida indoleacetic acid in development of the host plant root system. *Appl. Environ. Microbiol.* **68**, 3795–3801 (2002).

60. M. Espinosa-Urgel, A. Salido, J.-L. Ramos, Genetic analysis of functions involved in adhesion of Pseudomonas putida to seeds. *J. Bacteriol.* **182**, 2363–2369 (2000).

61. A. Ramette, M. Frapolli, M. F. L. Saux, C. Gruffaz, J. M. Meyer, G. Défago, L. Sutra, Y. Moënne-Loccoz, Pseudomonas protegens sp. nov., widespread plant-protecting bacteria producing the biocontrol compounds 2,4-diacetylphloroglucinol and pyoluteorin. *Syst. Appl. Microbiol.* **34**, 180–188 (2011).

62. D. Ercolini, A. Casaburi, A. Nasi, I. Ferrocino, R. di Monaco, P. Ferranti, G. Mauriello, F. Villani, Different molecular types of Pseudomonas fragi have the same overall behaviour as meat spoilers. *Int. J. Food Microbiol.* **142**, 120–131 (2010).

63. J. Lalucat, A. Bennasar, R. Bosch, E. García-Valdés, J. Palleroni Norberto, Biology of Pseudomonas stutzeri. *Microbiol. Mol. Biol. Rev.* **70**, 510–547 (2006).

64. U. Tattawasart, J. Y. Maillard, J. R. Furr, A. D. Russell, Development of resistance to chlorhexidine diacetate and cetylpyridinium chloride in Pseudomonas stutzeri and changes in antibiotic susceptibility. *J. Hosp. Infect.* **42**, 219–229 (1999).

65. L. Gardan, H. Shafik, S. Belouin, R. Broch, F. Grimont, P. A. Grimont, DNA relatedness among the pathovars of Pseudomonas syringae and description of Pseudomonas tremae sp. nov.

and Pseudomonas cannabina sp. nov. (ex Sutic and Dowson 1959). *Int. J. Syst. Bacteriol.* **49**, 469–478 (1999).

66. H. Y. Yun, H. Kim, First report of Pseudomonas amygdali causing bacterial leaf spot of Mallotus japonicus in South Korea. *For. Pathol.* **51**, e12707 (2021).

67. A. W. T. F. Chin, G. V. Bloemberg, I. H. Mulders, L. C. Dekkers, B. J. Lugtenberg, Root colonization by phenazine-1-carboxamide-producing bacterium Pseudomonas chlororaphis PCL1391 is essential for biocontrol of tomato foot and root rot. *Mol. Plant Microbe Interact.* **13**, 1340–1345 (2000).

68. J. M. Yu, D. Wang, L. S. Pierson 3rd, E. A. Pierson, Effect of producing different phenazines on bacterial fitness and biological control in Pseudomonas chlororaphis 30-84. *Plant Pathol. J.* **34**, 44–58 (2018).

69. S. W. Kwon, J. S. Kim, I. C. Park, S. H. Yoon, D. H. Park, C. K. Lim, S. J. Go, Pseudomonas koreensis sp. nov., Pseudomonas umsongensis sp. nov. and Pseudomonas jinjuensis sp. nov., novel species from farm soils in Korea. *Int. J. Syst. Evol. Microbiol.* **53**, 21–27 (2003).

70. G. F. Rafikova, T. Y. Korshunova, L. F. Minnebaev, S. P. Chetverikov, O. N. Loginov, A new bacterial strain, Pseudomonas koreensis IB-4, as a promising agent for plant pathogen biological control. *Microbiology* **85**, 333–341 (2016).

71. S. M. Kang, S. Asaf, A. L. Khan, Lubna, A. Khan, B. G. Mun, M. A. Khan, H. Gul, I. J. Lee, Complete genome sequence of Pseudomonas psychrotolerans CS51, a plant growth-promoting bacterium, under heavy metal stress conditions. *Microorganisms* **8**, 382 (2020).

72. Y. Li, D. Wang, S. Cao, X. Wang, G. Ren, First report of bacterial leaf spot on tobacco caused by Pseudomonas psychrotolerans in China. *Plant Dis.* **107**, 935 (2023).

73. A. A. Belimov, I. C. Dodd, V. I. Safronova, N. Hontzeas, W. J. Davies, Pseudomonas brassicacearum strain Am3 containing 1-aminocyclopropane-1-carboxylate deaminase can show both pathogenic and growth-promoting properties in its interaction with tomato. *J. Exp. Bot.* **58**, 1485–1495 (2007).

74. B. Dutta, R. Gitaitis, G. Agarwal, T. Coutinho, D. Langston, Pseudomonas coronafaciens sp. nov., a new phytobacterial species diverse from Pseudomonas syringae. *PLOS ONE* **13**, e0208271 (2018).

75. S. M. Bhatawadekar, Community-acquired urinary tract infection by pseudomonas oryzihabitans. *J. Glob. Infect. Dis.* **5**, 82–84 (2013).

76. Y. Hou, Y. Zhang, L. Yu, X. Ding, L. Liu, L. Wang, S. Huang, First report of Pseudomonas oryzihabitans causing rice panicle blight and grain discoloration in China. *Plant Dis.* **104**, 3055 (2020).

77. W. Adam, F. Heckel, C. R. Saha-Möller, M. Taupp, P. Schreier, A highly enantioselective biocatalytic sulfoxidation by the topsoil bacterium Pseudomonas frederiksbergensis. *Tetrahedron: Asymmetry* **15**, 983–985 (2004).

78. C. Ramos, I. M. Matas, L. Bardaji, I. M. Aragón, J. Murillo, Pseudomonas savastanoi pv. savastanoi: Some like it knot. *Mol. Plant Pathol.* **13**, 998–1009 (2012).

79. L. Tvrzová, P. Schumann, C. Spröer, I. Sedláček, Z. Páčová, O. Šedo, Z. Zdráhal, M. Steffen, E. Lang, Pseudomonas moraviensis sp. nov. and Pseudomonas vranovensis sp. nov., soil bacteria isolated on nitroaromatic compounds, and emended description of Pseudomonas asplenii. *Int. J. Syst. Evol. Microbiol.* **56**, 2657–2663 (2006).

80. M. Gennari, F. Dragotto, A study of the incidence of different fluorescent Pseudomonas species and biovars in the microflora of fresh and spoiled meat and fish, raw milk, cheese, soil and water. *J. Appl. Bacteriol.* **72**, 281–288 (1992).

81. S. Verhille, N. Baida, F. Dabboussi, D. Izard, H. Leclerc, Taxonomic study of bacteria isolated from natural mineral waters: Proposal of Pseudomonas jessenii sp. nov. and Pseudomonas mandelii sp. nov. *Syst. Appl. Microbiol.* **22**, 45–58 (1999).

82. R. Li, Y. Jiang, X. Wang, J. Yang, Y. Gao, X. Zi, X. Zhang, H. Gao, N. Hu, Psychrotrophic Pseudomonas mandelii CBS-1 produces high levels of poly-β-hydroxybutyrate. *Springerplus* **2**, 335 (2013).

83. M. Elomari, L. Coroler, S. Verhille, D. Izard, H. Leclerc, Pseudomonas monteilii sp. nov., isolated from clinical specimens. *Int. J. Syst. Bacteriol.* **47**, 846–852 (1997).

84. M. Tohya, S. Watanabe, K. Teramoto, K. Uechi, T. Tada, K. Kuwahara-Arai, T. Kinjo, S. Maeda, I. Nakasone, N. N. Zaw, S. Mya, K. N. Zan, H. H. Tin, J. Fujita, T. Kirikae, Pseudomonas asiatica sp. nov., isolated from hospitalized patients in Japan and Myanmar. *Int. J. Syst. Evol. Microbiol.* **69**, 1361–1368 (2019).

85. D. Kakembo, Y. H. Lee, Analysis of traits for biocontrol performance of Pseudomonas parafulva JBCS1880 against bacterial pustule in soybean plants. *Biol. Control* **134**, 72–81 (2019).

86. U. Behrendt, A. Ulrich, P. Schumann, Fluorescent pseudomonads associated with the phyllosphere of grasses; Pseudomonas trivialis sp. nov., Pseudomonas poae sp. nov. and Pseudomonas congelans sp. nov. *Int. J. Syst. Evol. Microbiol.* **53**, 1461–1469 (2003).

87. M. von Neubeck, C. Huptas, C. Glück, M. Krewinkel, M. Stoeckel, T. Stressler, L. Fischer, J. Hinrichs, S. Scherer, M. Wenning, Pseudomonas helleri sp. nov. and Pseudomonas weihenstephanensis sp. nov., isolated from raw cow's milk. *Int. J. Syst. Evol. Microbiol.* **66**, 1163–1173 (2016).

88. P. Ioannou, G. Vougiouklakis, A systematic review of human infections by Pseudomonas mendocina. *Trop. Med. Infect. Dis.* **5**, 71 (2020).

89. C. O. Onwosi, F. J. Odibo, Effects of carbon and nitrogen sources on rhamnolipid biosurfactant production by Pseudomonas nitroreducens isolated from soil. *World J. Microbiol. Biotechnol.* **28**, 937–942 (2012).

90. A. Novinscak, M. Filion, Long term comparison of talc- and peat-based phytobeneficial Pseudomonas fluorescens and Pseudomonas synxantha bioformulations for promoting plant growth. *Front. Sustain. Food Syst.* **4**, e602911 (2020).
